# From disease-specific control to system-embedded governance: a multidimensional policy design analysis of stroke prevention and stroke center development in China

**DOI:** 10.3389/fpubh.2026.1888583

**Published:** 2026-08-10

**Authors:** Xingchen Qiu, Bo Wang, Jun Sun, Shuang Pei, Zaihang Zhang, Shiqian Guo, Ning Wang, Jing Zhou, Bing Guo, Shidun Chen, Changming Wen

**Affiliations:** 1 Department of Neurology, Nanyang Central Hospital, Nanyang, China; 2 National Clinical Key Specialty & National Demonstration Advanced Stroke Center, Nanyang Central Hospital, Nanyang, China; 3 Shenzhen PingLe Orthopedic Hospital Affiliated to Guangzhou University of Chinese Medicine, Shenzhen, China

**Keywords:** health-system governance, policy instruments, policy text analysis, stroke center, stroke prevention

## Abstract

**Background:**

Stroke remains a major cause of death, disability, and long-term care burden in China. National policy has expanded from disease-specific prevention and treatment to broader arrangements involving chronic disease governance, emergency care, hierarchical services, rehabilitation, and medical quality improvement. However, the policy design and governance structure remain underexamined.

**Methods:**

We conducted a multidimensional national policy text analysis of stroke prevention and stroke center development in China. The final corpus comprised 33 national policies issued between 2009 and 2026, including 11 Main policies and 22 Extended policies. Policies were classified by document function, and 1,739 atomic meaning units were coded for policy instruments, governance tasks, strict and contextual actors, policy objects, and policy integration. A stratified sample of 348 units was independently coded, followed by consensus adjudication.

**Results:**

The corpus showed a descriptive three-stage progression from early disease-specific screening and quality-control arrangements, through system expansion, to system-embedded and quality-oriented governance. The final adjudicated dataset contained 16,882 code assignments. Among 5,263 policy-instrument assignments, environment-side instruments accounted for 47.0%, supply-side instruments for 32.2%, and demand-side instruments for 20.7%. Standardized diagnosis, treatment, and acute care was the most frequent governance task, followed by quality improvement, system coordination, and risk-factor control. Hospitals, medical institutions, and stroke centers were the most frequently specified strict actors, while other actor groups showed differentiated task profiles. Policy integration connected stroke governance with medical quality and safety, specialty capacity, chronic disease governance, public health, emergency care, rehabilitation, referral, and financing. Cohen’s kappa across the six coding dimensions ranged from 0.595 to 0.746.

**Conclusion:**

China’s national stroke policy has evolved toward system-embedded, task-differentiated, and quality-oriented governance. Future policy design may benefit from stronger demand-side and continuity-oriented instruments, clearer cross-setting responsibilities, and closer alignment between stroke-specific policies and wider health-system arrangements.

## Introduction

Stroke remains one of the leading causes of death and disability worldwide, and it poses a particularly substantial challenge in China because of the country’s large population, high disease burden, regional variation in service capacity, and the coexistence of prevention, acute care, rehabilitation, and chronic management needs (1, 2). Beyond its direct clinical consequences, stroke generates broader social and health-system pressures through long-term disability, impaired health-related quality of life, caregiver burden, and continuing needs for rehabilitation, secondary prevention, and community-based follow-up (3–5). For that reason, stroke governance extends beyond disease treatment to include risk-factor control, early identification, standardized diagnosis and treatment, emergency referral, rehabilitation continuity, quality improvement, and regional coordination across levels of care (6–8). Recent work on integrated stroke care also emphasizes the need to connect acute treatment with post-acute management and patient-centered continuity of care (9). Thus, stroke governance requires coordinated policy arrangements across prevention, emergency care, hospital treatment, rehabilitation, long-term follow-up, and quality improvement.

In China, the policy importance of stroke has become increasingly visible over the past two decades. The policy agenda has expanded from early concerns with high-risk population screening, technical standardization, and clinical quality control to a broader system of stroke prevention and control that includes stroke center development, acute care capacity improvement, hierarchical diagnosis and treatment, emergency response, rehabilitation continuity, and integration into wider chronic disease governance (6–8, 10, 11). Stroke center development, in particular, has emerged as a key institutional arrangement for translating national policy objectives into service-delivery and quality-governance practices. As an organizational platform, the stroke center links acute-phase diagnosis and treatment, in-hospital workflow optimization, multidisciplinary collaboration, quality monitoring, data reporting, and regional coordination. It therefore occupies an important position at the intersection of disease-specific management, service-capacity building, and broader health-system strengthening (8, 12).

The policy field surrounding stroke in China is also distinctive because it is shaped by both disease-specific documents and a wider institutional environment that includes national chronic disease strategies, Healthy China initiatives, emergency care policies, rehabilitation planning, specialty capacity development, hierarchical diagnosis and treatment, health-financing arrangements, and national medical quality and safety improvement (13–15). Stroke prevention and stroke center development are therefore governed through a direct stroke-policy layer and a broader supporting policy layer. This structure is analytically important but methodologically challenging. A narrow focus on stroke-specific documents may underestimate the contribution of wider health-system policies, whereas an overly broad inclusion strategy may dilute the disease-specific logic of stroke governance. A two-layer corpus with explicit relevance rules is therefore needed to preserve both topic specificity and institutional context.

Existing studies on stroke in China have largely focused on epidemiological burden, clinical pathways, service capacity (16, 17), center certification, and health outcomes (18, 19), while policy-related work has more often taken the form of systematic reviews, descriptive summaries, or discussions of implementation challenges (2, 20, 21). To our knowledge, systematic policy text analyses focusing specifically on the national policy configuration of stroke prevention and stroke center development in China remain limited. In particular, less is known about how document functions, policy instruments, governance tasks, responsible actors, policy objects, and broader health-system integration have been configured across stages, and what governance logic can be inferred from their relationships. More generally, policy text analyses in the health field in China have demonstrated the value of examining instrument distribution, policy emphases, and institutional change, but stroke-related policy remains underexamined from this perspective (22–24). This gap matters because policy design affects what is prioritized, who is expected to act, which populations or system components are targeted, and how institutional supports, regulatory expectations, and service-delivery pathways are assembled.

A multidimensional policy design analysis is particularly useful in this context. Conceptually, document functions locate policies within the national policy architecture; policy instruments represent the means of intervention; governance tasks represent the substantive ends pursued; responsible actors indicate who is expected to act; policy objects identify who or what is targeted; and policy-integration domains capture the wider health-system context. Examining these dimensions together links policy position, means, ends, responsibility, targets, and system embedding. Separating explicitly stated actors from actors inherited under the predefined contextual rule further clarifies responsibility allocation without relying on institutional assumptions, while distinguishing responsible actors from policy objects avoids treating recipients of screening, treatment, education, or regulation as implementing bodies (25–27).

To address this gap, the present study conducted a multidimensional national policy text analysis of stroke prevention and stroke center development in China. It used a two-layer corpus that distinguished directly stroke-focused national policies from broader policies containing explicitly traceable stroke-relevant provisions. Drawing on document-function analysis, policy-instrument analysis, directed thematic analysis, and complementary analyses of governance tasks, responsible actors, policy objects, and policy integration, the study examined policy evolution, instrument configuration, task orientation, responsibility allocation, target orientation, and system-level embedding. It addressed four questions: how the national policy framework evolved over time; what policy instruments were used and how their configuration changed across stages; how governance tasks, responsible actors, and policy objects were aligned; and how stroke-specific arrangements were integrated into the wider health-system context.

## Methods

### Study design and document identification

We conducted a longitudinal, directed quantitative content analysis of national policy documents concerning stroke prevention and control, stroke-center development, acute and rehabilitation services, and the wider health-system arrangements supporting these activities in China. The unit of analysis was the formal national policy document, with meaning units used for within-document coding.

Candidate documents were identified from official websites of the State Council, the National Health Commission and other central government departments. Searches were cross-checked against PKULAW and the National Database of Laws and Regulations, and were supplemented by tracing official attachments, cited policies, linked implementation documents and related national plans. Search terms combined disease-specific and system-level concepts, including stroke, cerebrovascular disease, stroke center, screening, prevention and control, diagnosis and treatment, emergency care, rehabilitation, hierarchical diagnosis and treatment, chronic disease, medical quality, specialty capacity and health financing.

**Figure 1:**
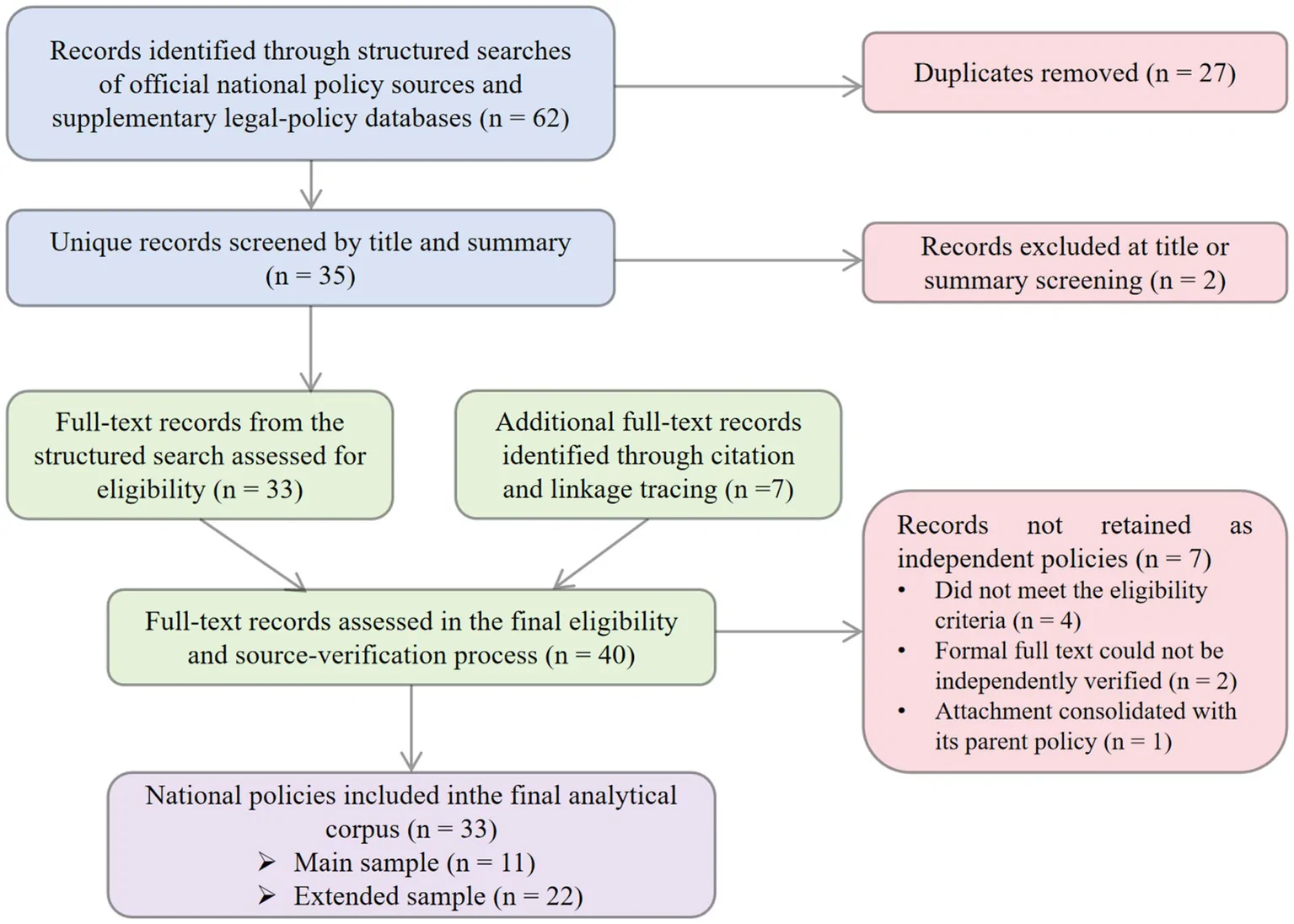
Study flow of policy identification, eligibility assessment, source verification, and final inclusion. The structured search identified 62 records. After removal of 27 duplicates, 35 unique records were screened, of which two were excluded at title or summary review. Thirty-three full-text records from the structured search and seven additional records identified through citation and linkage tracing underwent final eligibility assessment and source verification. Four records did not meet the eligibility criteria, two lacked independently verifiable formal full text, and one attachment was consolidated with its parent policy. The final analytical corpus comprised 33 national policies, including 11 main policies and 22 extended policies.

Documents were eligible when they were formal national-level policy texts with normative or operational content and either directly addressed stroke-related prevention, diagnosis, treatment, rehabilitation or quality governance, or contained provisions with an explicit and traceable effect on the stroke care pathway or its enabling health-system environment. News reports, publicity materials, commentaries, documents without normative content, duplicate records and texts lacking substantive relevance were excluded. The identification process yielded 62 records. After removal of 27 duplicates, 35 unique records were screened; two were excluded at title or summary review and 33 full texts were assessed. Four did not meet the eligibility criteria. Citation and linkage tracing identified seven additional eligible policies. Two records without independently verifiable formal full text were excluded, and one attachment was treated as part of its parent policy rather than as a separate document. The final corpus comprised 33 policies issued between 2009 and 2026 (Figure 1).

### Corpus structure and text preparation

To preserve both disease specificity and institutional context, the corpus was organized into two analytically linked samples. The Main sample comprised 11 policies directly focused on stroke, cerebrovascular disease, stroke centers, screening, standardized care, rehabilitation, or stroke-specific quality governance. The Extended sample comprised 22 broader policies addressing chronic disease governance, emergency care, referral, rehabilitation, specialty capacity, medical quality, public health, or financing. Extended-policy clauses were retained when they met at least one of four predefined criteria: explicit reference to stroke or cerebrovascular disease; direct relevance to prevention, emergency care, diagnosis and treatment, rehabilitation, referral, or continuity along the stroke pathway; identifiable governance or capacity implications for stroke-related services; or financing and payment arrangements with a clearly traceable application to stroke care. General reform statements, administrative boilerplate, definitions, and macro-level provisions without an identifiable stroke pathway, service, governance, or financing link were excluded.

Official full texts were used wherever available. Each retained passage was linked to its policy identifier and section or article location. Text preparation was limited to correction of non-substantive extraction artifacts, such as duplicated spaces, line breaks, page headers and isolated layout symbols; wording was otherwise preserved verbatim. Policies were grouped into three periods for temporal comparison: S1 (2009–2014), S2 (2015–2020) and S3 (2021–2026).

### Meaning-unit segmentation and coding framework

Policies were segmented into atomic meaning units before coding. A meaning unit was defined as the smallest verbatim segment that expressed a distinct policy action, responsibility, instrument, governance task, target or integration relationship while retaining sufficient context for interpretation. Clauses containing several independently actionable components were split, whereas closely connected predicates serving a single action were retained together. Headings, article numbers and immediately controlling text were preserved as locators or context rather than rewritten into the unit. The segmentation and coding rules were refined through pilot coding of 62 units selected to maximize variation across sample type, stage and policy domain. Application of the final rules produced 1,739 meaning units.

The coding framework contained 55 codes. At the document level, seven document-function categories described strategic planning, administrative deployment, technical guidance, institutional construction, service-system arrangements, quality governance and target setting, and financing or payment support. Up to two functions were assigned when a policy had clearly separable principal roles.

Meaning units were coded across six fields. Policy instruments comprised 14 codes grouped into supply-side, environment-side and demand-side instruments. Governance tasks comprised seven categories: risk-factor control and high-risk screening; standardized diagnosis, treatment and acute care; stroke-center development; system building and coordination; quality control and performance improvement; rehabilitation and continuity of care; and population prevention and health promotion. Responsible actors were classified into ten categories and recorded separately as strict and contextual assignments. Strict actors had to be explicitly named as action-bearing subjects in the current unit. Contextual actors could be inherited only from the same natural paragraph, numbered provision or immediately adjacent sentence when the actor had already been explicitly identified. Policy objects were coded separately from responsible actors and comprised nine target categories. Eight policy-integration domains captured links to chronic disease governance, emergency care, rehabilitation and continuity, hierarchical care and referral, specialty and stroke-center capacity, public health and population prevention, medical quality and safety, and health financing and payment.

Coding was multi-label. More than one code could be assigned within a dimension when each code was independently supported by the text, and no code was forced when a dimension was absent. Boundary rules distinguished institution building from coordination, action-bearing actors from policy recipients or institutional objects, direct fiscal input from payment arrangements, and disease-specific integration from generic health-system reform. Operational definitions, inclusion and exclusion criteria, and key boundary rules for all 55 codes are provided in Appendix Table A7.

### Coding procedure

One researcher coded the complete corpus using the finalized coding manual. Each coding record contained the verbatim meaning unit, necessary contextual text, source location and all applicable code assignments. Units involving contextual-actor inheritance, lower coding confidence, text normalization or dense multi-label coding were flagged for additional review. The full dataset was checked for duplicate records, invalid codes, discontinuous identifiers and consistency between unit-level and assignment-level files before reliability analysis.

### Independent coding, agreement, and adjudication

A second researcher, blinded to the primary coding, independently coded a prespecified sample of 348 meaning units, representing 20.0% of the corpus and covering all 33 policies. Each policy contributed approximately 20% of its units. Within policies, the sample was stratified between units flagged for additional review and standard units, and a reproducible computer-generated ordering was used for selection. The sample covered the Main and Extended samples, all three stages and the principal policy domains.

The second researcher also independently assigned document functions to all 33 policies and reviewed the relevance classification of all 845 retained Extended-sample units using four criteria: explicit disease relevance, direct pathway relevance, governance relevance and financing or payment relevance. The second coder received the source text and coding instructions but not the primary assignments. Units flagged for additional review but not included in the independent sample were examined jointly after independent coding and were not included in agreement estimates.

For each multi-label dimension, coder decisions were converted to unit-by-code binary matrices. Agreement was summarized using exact-set agreement, mean and median Jaccard similarity, micro-averaged precision, recall and F1, and Cohen’s kappa across binary decisions. Ninety-five percent confidence intervals were estimated using 1,000 policy-clustered bootstrap samples. Document-function agreement was assessed using raw agreement and Cohen’s kappa for the primary function and exact agreement for the complete function set. Extended-sample relevance agreement was assessed using exact profile agreement, micro-averaged F1 and criterion-specific agreement. An inclusion-versus-exclusion kappa was not calculated because the independently reviewed Extended set contained retained units only.

All disagreements were resolved by the two coders through direct comparison with the original policy text, relevant context and the coding rules. Multi-coding was retained only when each assignment had independent textual support. The adjudicated decisions were incorporated into the complete corpus, and the resulting consensus-coded dataset was used for all analyses.

### Quantitative analysis

Analyses distinguished policy counts, meaning-unit counts and assignment counts. Policy prevalence was the proportion of policies within the relevant overall, stage or sample denominator containing at least one instance of a code. Unit prevalence was the proportion of meaning units containing a code. Assignment counts represented all retained code assignments; because coding was multi-label, assignment-based or code-prevalence percentages could exceed 100% when summed across categories.

Temporal comparisons used S1, S2 and S3. Strict actors were used as the primary measure of responsibility allocation, while contextual and combined actor results were reported as supplementary analyses. Combined actors represented the union of strict and contextual assignments rather than their arithmetic sum. Actor–task and instrument–task co-occurrences were calculated at the meaning-unit level, with each code pair counted no more than once per unit. Policy integration was reported as both meaning-unit prevalence and policy coverage. Main analyses were descriptive and were conducted using structured data files and R version 4.5.1. Because multi-label assignments were nested within policies, assignment-level chi-square tests were not used. As a policy-level sensitivity analysis, each of the 33 policies was treated as an independent unit. Counts of supply-side, environment-side, and demand-side assignments were converted to within-policy compositions; a 0.5 pseudocount was added before centered log-ratio transformation. The global stage effect was evaluated using a pseudo-F statistic with 99,999 permutations of stage labels, and R^2^ quantified explained compositional variation. Robustness checks used untransformed proportions, a permutation test of multivariate dispersion, and category-specific Kruskal–Wallis tests with Holm adjustment. The script and policy-level input data are available from the corresponding author on reasonable request.

## Results

### Characteristics and temporal distribution of the policy corpus

The final corpus comprised 33 national policy documents issued between 2009 and 2026, including 11 Main policies and 22 Extended policies. Rule-based segmentation produced 1,739 atomic meaning units, of which 894 were derived from the Main sample and 845 from the Extended sample. These units received 16,882 code assignments across the six meaning-unit coding dimensions (Table 1).

**Table 1:** Composition of the final analytical corpus.

Stratification	Category	Policies, *n* (%)	Meaning units, *n* (%)	Code assignments, *n*
Sample group	Extended	22 (66.7%)	845 (48.6%)	7,113
Sample group	Main	11 (33.3%)	894 (51.4%)	9,769
Analytical stage	S1 (2009–2014)	6 (18.2%)	150 (8.6%)	1,181
Analytical stage	S2 (2015–2020)	13 (39.4%)	378 (21.7%)	3,487
Analytical stage	S3 (2021–2026)	14 (42.4%)	1,211 (69.6%)	12,214
Overall	Final corpus	33 (100.0%)	1,739 (100.0%)	16,882

Policy percentages use all 33 included policies as the denominator, and meaning-unit percentages use all 1,739 atomic meaning units. Code-assignment counts include all retained assignments across the six meaning-unit coding dimensions. Because coding was multi-label, the number of assignments exceeds the number of meaning units. Main policies directly addressed stroke-specific prevention, care, rehabilitation, stroke-center development, or quality governance; Extended policies contributed only clauses with an explicit or clearly traceable relationship to the stroke pathway or its enabling health-system environment.

**Figure 2:**
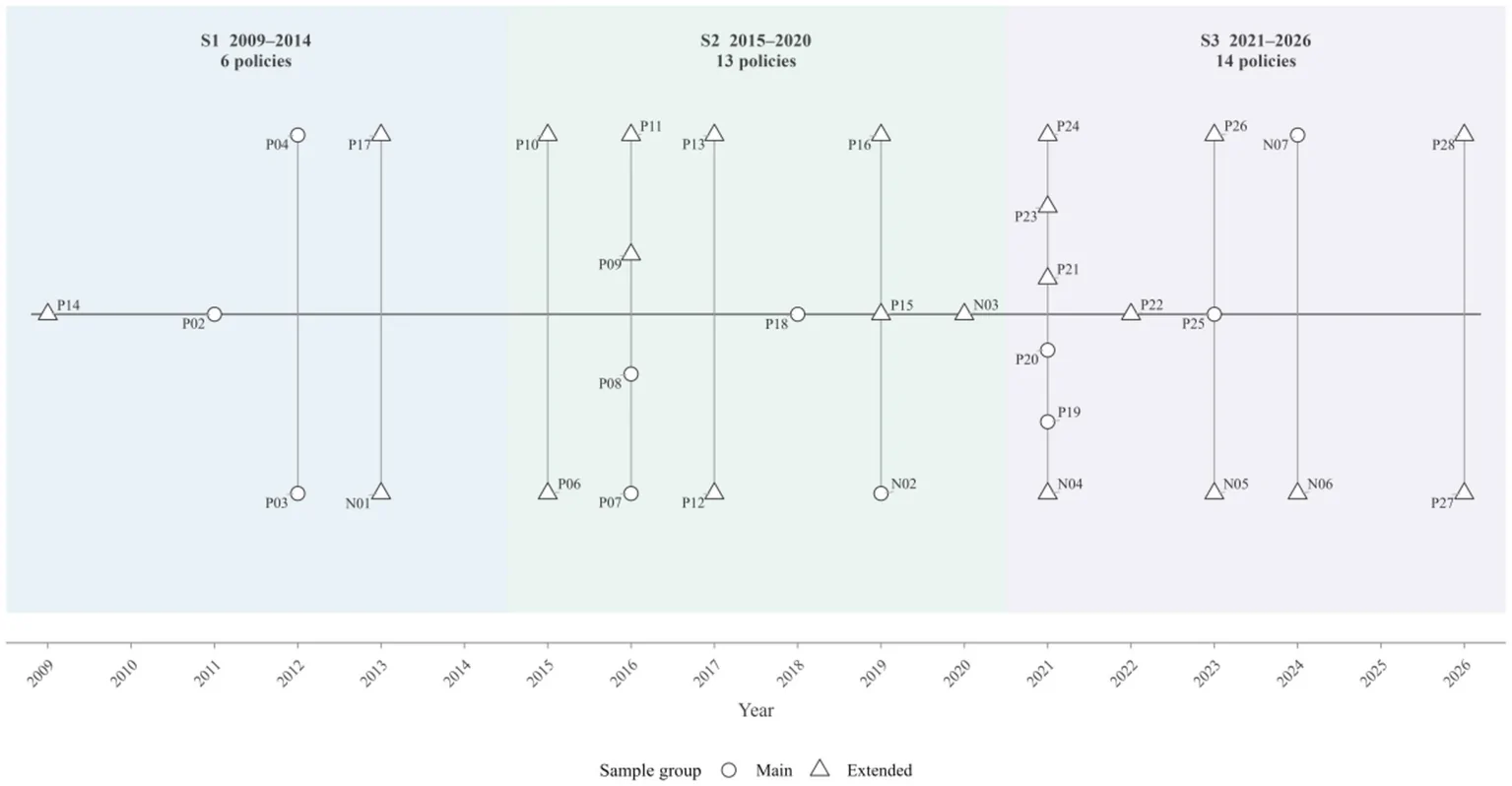
Timeline and stage distribution of the final national policy corpus. Each point represents one included policy. Point shape distinguishes Main policies, which directly addressed stroke-specific prevention, care, rehabilitation, stroke-center development, or quality governance, from extended policies, which contained explicitly traceable stroke-relevant provisions within broader health-system arrangements. Background shading denotes S1 (2009–2014), S2 (2015–2020), and S3 (2021–2026). The final stage distribution was 6, 13, and 14 policies, respectively.

The temporal distribution was uneven. S1 (2009–2014) contained 6 policies, 150 meaning units, and 1,181 assignments; S2 (2015–2020) contained 13 policies, 378 meaning units, and 3,487 assignments; and S3 (2021–2026) contained 14 policies, 1,211 meaning units, and 12,214 assignments. Thus, S3 accounted for 69.6% of all meaning units and 72.3% of all assignments. The policy timeline illustrates the expansion of both the Main and Extended policy layers across the three stages (Figure 2).

At the policy level, prevention and screening was the most frequent primary domain (11/33), followed by quality and stroke-center governance (9/33), system, referral, and capacity arrangements (8/33), emergency and acute care (3/33), and rehabilitation and continuity of care (2/33). Strategic planning was the most frequent primary document function (12/33), followed by administrative deployment (6/33), technical guidance (5/33), institutional construction (4/33), quality governance and target setting (3/33), and service-system arrangements (3/33). When primary and secondary functions were considered together, service-system arrangements occurred in 18 policies and administrative deployment in 14. Selected policy-level characteristics, including abbreviated policy title, year, sample group and stage, primary domain, document functions, and number of retained meaning units, are summarized in Table 2. Full Chinese titles, English translations, issuing authorities, and official sources are provided in Appendix Table A1.

**Table 2:** Selected characteristics of the included national policy documents.

Policy	Year	Sample/stage	Domain	Functions	Units
P14. Medical Quality Control Centers (Trial)	2009	Ext./S1	Quality/center	DF-06; DF-04	19
P02. Standardized Stroke Care and Quality Control	2011	Main/S1	Quality/center	DF-02; DF-06	21
P03. High-Risk Stroke Screening and Intervention Pilot	2012	Main/S1	Prevention/screening	DF-02; DF-03	48
P04. Stroke Screening and Prevention Bases	2012	Main/S1	Quality/center	DF-04; DF-02	6
N01. Referral Standards for Stroke Rehabilitation	2013	Ext./S1	Rehabilitation/continuity	DF-03; DF-05	6
P17. Prehospital Emergency Medical Care	2013	Ext./S1	Emergency/acute care	DF-05; DF-06	50
P06. Acute Cardiovascular and Cerebrovascular Treatment Capacity	2015	Ext./S2	Emergency/acute care	DF-03; DF-05	38
P10. Tiered Diagnosis and Treatment System	2015	Ext./S2	System/referral/capacity	DF-01; DF-05	28
P07. Hospital Stroke Center Development and Management	2016	Main/S2	Quality/center	DF-04; DF-03	42
P08. Comprehensive Stroke Prevention and Control Plan	2016	Main/S2	Prevention/screening	DF-01; DF-02	46
P09. Healthy China 2030	2016	Ext./S2	Prevention/screening	DF-01; DF-05	20
P11. 13th Five-Year Health Plan	2016	Ext./S2	System/referral/capacity	DF-01; DF-05	21
P12. Chronic Disease Prevention and Treatment Plan (2017–2025)	2017	Ext./S2	Prevention/screening	DF-01; DF-05	32
P13. Medical Service Improvement Action Plan (2018–2020)	2017	Ext./S2	System/referral/capacity	DF-02; DF-05	22
P18. Management of Stroke Diagnosis and Treatment	2018	Main/S2	Quality/center	DF-02; DF-04	18
N02. Stroke Screening and Comprehensive Intervention Plan	2019	Main/S2	Prevention/screening	DF-03; DF-06	42
P15. State Council Opinions on the Healthy China Initiative	2019	Ext./S2	Prevention/screening	DF-01; DF-02	13
P16. Healthy China Initiative (2019–2030)	2019	Ext./S2	Prevention/screening	DF-01; DF-05	26
N03. Prehospital Emergency Medical Services	2020	Ext./S2	Emergency/acute care	DF-05; DF-02	30
N04. Rehabilitation Medical Services	2021	Ext./S3	Rehabilitation/continuity	DF-01; DF-05	25
P19. Strengthening Stroke Prevention and Reducing New Disabilities	2021	Main/S3	Prevention/screening	DF-02; DF-05	31
P20. Chinese Stroke Prevention and Treatment Guidelines (2021)	2021	Main/S3	Prevention/screening	DF-03; DF-06	431
P21. 14th Five-Year National Development Plan and 2035 Objectives	2021	Ext./S3	System/referral/capacity	DF-01; DF-05	25
P23. 14th Five-Year Clinical Specialty Capacity Plan	2021	Ext./S3	System/referral/capacity	DF-01; DF-04	54
P24. Thousand-County Hospital Capacity Project (2021–2025)	2021	Ext./S3	System/referral/capacity	DF-02; DF-05	44
P22. 14th Five-Year National Health Plan	2022	Ext./S3	System/referral/capacity	DF-01; DF-05	116
N05. Medical Quality Control Centers	2023	Ext./S3	Quality/center	DF-06; DF-04	101
P25. Expert Committee for the Stroke Disability-Reduction Project	2023	Main/S3	Quality/center	DF-04; DF-02	11
P26. Cardiovascular and Cerebrovascular Disease Action Plan (2023–2030)	2023	Ext./S3	Prevention/screening	DF-01; DF-05	89
N06. Provincial Technical Lead Institutions for Cardiovascular and Cerebrovascular Disease Control	2024	Ext./S3	Quality/center	DF-04; DF-02	13
N07. Cerebrovascular Disease Prevention and Control Guidelines (2024)	2024	Main/S3	Prevention/screening	DF-03; DF-05	198
P27. Measures for Accelerating Tiered Diagnosis and Treatment	2026	Ext./S3	System/referral/capacity	DF-05; DF-02	64
P28. National Medical Quality and Safety Targets (2026)	2026	Ext./S3	Quality/center	DF-06; DF-02	9

Policy titles are shortened for display; full English translations of the official Chinese titles and source details are provided in Appendix Table A1. Ext. denotes the Extended sample. S1, S2, and S3 denote 2009–2014, 2015–2020, and 2021–2026, respectively. Domain indicates the primary policy domain. Functions are shown as document-function codes: DF-01, strategic planning; DF-02, administrative deployment; DF-03, technical guidance; DF-04, institutional construction; DF-05, service-system arrangement; DF-06, quality governance and target setting; and DF-07, financing or payment support. When two codes are shown, the first is the primary function and the second is the secondary function. Units indicates the number of retained atomic meaning units.

### Policy-instrument configuration and temporal change

**Figure 3:**
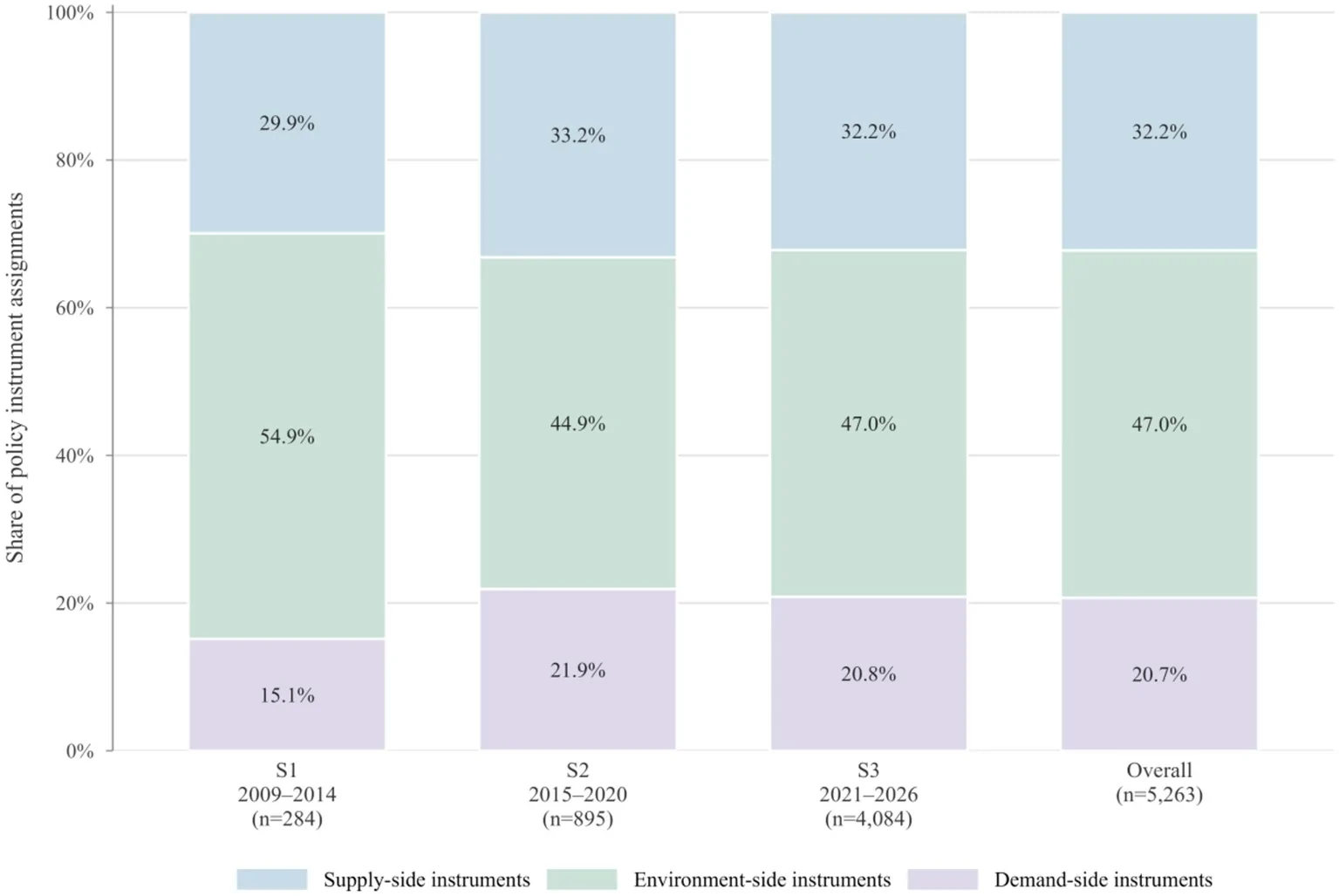
Distribution of major policy instrument categories across stages. Stacked bars show the percentage distribution of policy-instrument assignments within each stage and in the full corpus. Supply-side, environment-side, and demand-side instruments were aggregated from the 14 detailed instrument codes. Percentages sum to 100% within each bar. The *n* shown below each bar is the total number of policy-instrument assignments in that stage, not the number of meaning units.

Across the corpus, 5,263 policy-instrument assignments were retained. Environment-side instruments accounted for 2,476 assignments (47.0%), supply-side instruments for 1,697 (32.2%), and demand-side instruments for 1,090 (20.7%) (Figure 3). Environment-side instruments remained the largest category in each stage, accounting for 54.9% of instrument assignments in S1, 44.9% in S2, and 47.0% in S3. Supply-side instruments represented 29.9, 33.2, and 32.2%, respectively, while demand-side instruments increased from 15.1% in S1 to 21.9% in S2 and remained at 20.8% in S3.

At the policy level, the global stage-composition test was not statistically significant (pseudo-*F* = 1.899, R^2^ = 0.112, *p* = 0.147). Results were similar using untransformed within-policy proportions (pseudo-*F* = 2.174, R^2^ = 0.127, *p* = 0.089), and the multivariate dispersion check was non-significant (*p* = 0.183). None of the category-specific stage tests remained significant after Holm correction (adjusted *p* = 0.109–0.198; Appendix Table A8). The descriptive stage shifts should therefore be interpreted as corpus-level patterns rather than robust policy-level differences.

The meaning-unit analysis showed a related but distinct pattern because units could receive multiple instrument codes. Environment-side instruments occurred in 82.0% of S1 units, 76.5% of S2 units, and 83.6% of S3 units. Supply-side instruments occurred in 54.0, 53.2, and 66.6%, respectively. Demand-side instruments showed the largest increase, from 26.0% of S1 units to 43.1% of S2 units and 51.0% of S3 units. All three major instrument categories appeared in every S2 and S3 policy; demand-side instruments were present in five of the six S1 policies (Table 3).

**Table 3:** Policy instruments and governance tasks by stage.

Characteristic	S1		S2		S3	
	Unit *n* (%)	Policies *n*/*N*	Unit *n* (%)	Policies *n*/*N*	Unit *n* (%)	Policies *n*/*N*
Policy instruments						
Supply-side instruments	81 (54.0)	6/6	201 (53.2)	13/13	806 (66.6)	14/14
Environment-side instruments	123 (82.0)	6/6	289 (76.5)	13/13	1,013 (83.6)	14/14
Demand-side instruments	39 (26.0)	5/6	163 (43.1)	13/13	618 (51.0)	14/14
Governance tasks						
Risk-factor control and high-risk screening	35 (23.3)	3/6	107 (28.3)	12/13	337 (27.8)	9/14
Standardized diagnosis, treatment, and acute care	25 (16.7)	4/6	119 (31.5)	13/13	662 (54.7)	12/14
Stroke-center development	7 (4.7)	2/6	32 (8.5)	8/13	48 (4.0)	7/14
System building and coordination	30 (20.0)	4/6	128 (33.9)	13/13	399 (32.9)	14/14
Quality control and performance improvement	74 (49.3)	5/6	104 (27.5)	13/13	412 (34.0)	14/14
Rehabilitation and continuity of care	10 (6.7)	2/6	74 (19.6)	11/13	186 (15.4)	11/14
Population prevention and health promotion	7 (4.7)	2/6	48 (12.7)	12/13	161 (13.3)	12/14

Unit-level values are *n* (%) of meaning units within each stage; policy coverage is the number of policies containing at least one relevant unit divided by the total number of policies in that stage. Denominators were 150 units and 6 policies for S1, 378 units and 13 policies for S2, and 1,211 units and 14 policies for S3. S1, S2, and S3 denote 2009–2014, 2015–2020, and 2021–2026, respectively. Categories were non-exclusive.

At the detailed-code level, standards, norms, and procedures were most frequent, occurring in 763 units (43.9%) and all 33 policies. Institution, platform, and network building and screening, risk identification, and early intervention each occurred in 484 units (27.8%). Quality monitoring, evaluation, and improvement occurred in 469 units (27.0%), and technical and professional support in 456 (26.2%) (Appendix Figure A1). The Main sample contained higher unit prevalence of screening and early intervention (46.6% vs. 7.9%), standards and procedures (58.7% vs. 28.2%), technical support (36.1% vs. 15.7%), and quality monitoring (35.0% vs. 18.5%) than the Extended sample. In contrast, institution, platform, and network building was more prevalent in Extended units (39.5% vs. 16.8%) (Appendix Figure A2). Across stages, technical and professional support increased from 8.7% of S1 units to 32.5% of S3 units, while screening, risk identification, and early intervention increased from 14.7 to 33.9% (Appendix Table A2).

### Governance tasks and instrument-task alignment

Standardized diagnosis, treatment, and acute care was the most frequent governance task, occurring in 806 meaning units (46.3%). It was followed by quality control and performance improvement in 590 units (33.9%), system building and coordination in 557 (32.0%), risk-factor control and high-risk screening in 479 (27.5%), rehabilitation and continuity of care in 270 (15.5%), population prevention and health promotion in 216 (12.4%), and stroke-center development in 87 (5.0%).

Task prevalence varied across stages. Standardized diagnosis, treatment, and acute care increased from 16.7% of S1 units to 31.5% of S2 units and 54.7% of S3 units. System building and coordination increased from 20.0 to 33.9% and remained at 32.9% in S3. Rehabilitation and continuity of care rose from 6.7% in S1 to 19.6% in S2 and accounted for 15.4% of S3 units. Risk-factor control and high-risk screening remained comparatively stable at 23.3, 28.3, and 27.8%. Quality control and performance improvement was most prevalent in S1 (49.3%), declined in S2 (27.5%), and accounted for 34.0% of S3 units. Population prevention and health promotion increased from 4.7 to 12.7 and 13.3%, respectively. Stroke-center development remained less frequent at the unit level but was present in 2/6 S1 policies, 8/13 S2 policies, and 7/14 S3 policies (Table 3; Appendix Figure A3).

**Figure 4:**
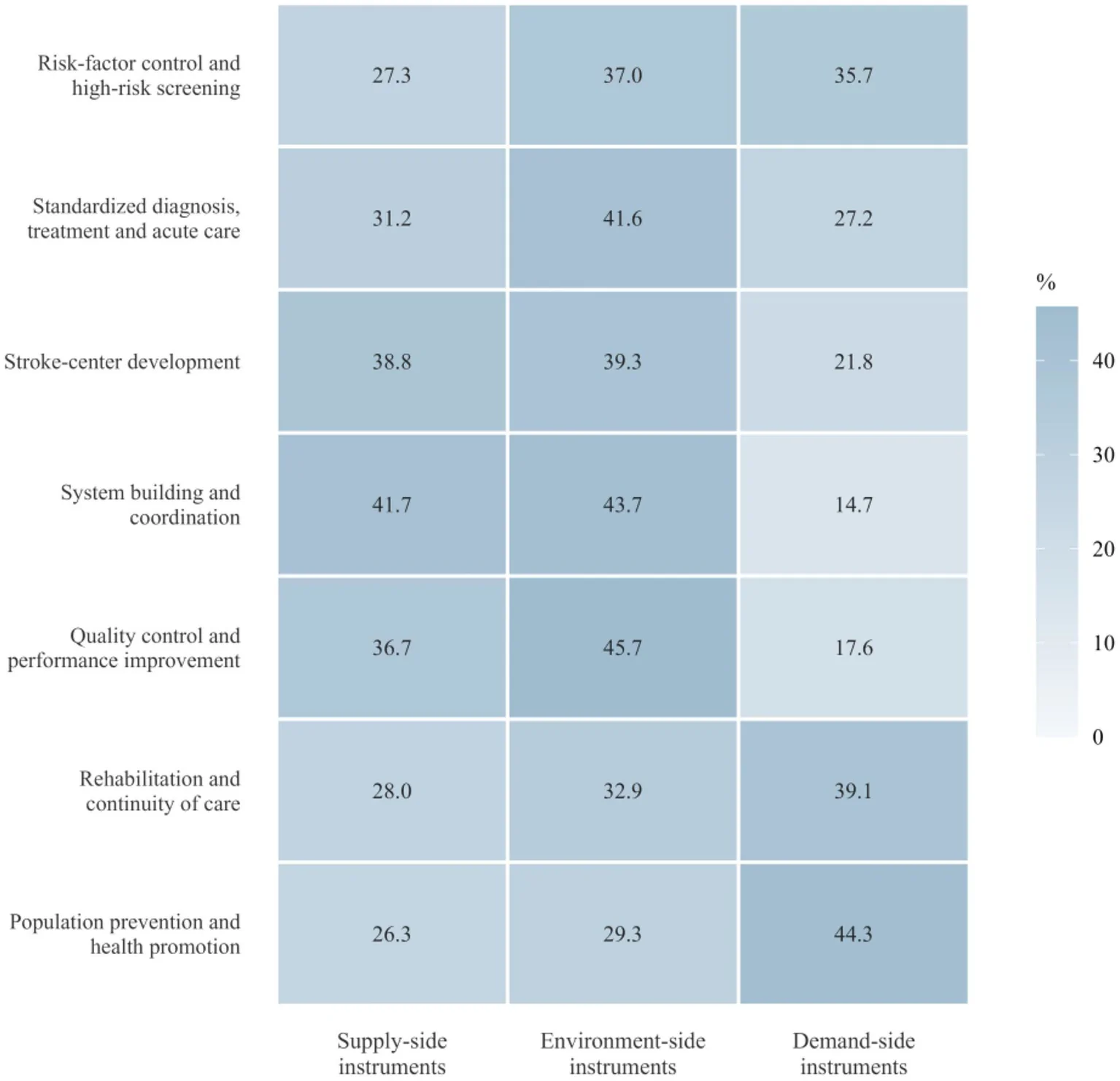
Alignment between governance tasks and major policy instrument categories. Cells show the percentage distribution of supply-side, environment-side, and demand-side instrument pairs within each governance task. Each task–instrument major-category pair was counted no more than once per meaning unit. Percentages are normalized within governance task and therefore sum to 100% across each row. A meaning unit could contribute to more than one governance task and more than one instrument category.

The instrument-task matrix showed differentiated instrument mixes across tasks (Figure 4; Appendix Table A3). Environment-side instruments formed the largest share of pairs for standardized diagnosis, treatment, and acute care (41.6%), stroke-center development (39.3%), system building and coordination (43.7%), and quality control and performance improvement (45.7%). Supply-side instruments also contributed substantially to system building and coordination (41.7%) and stroke-center development (38.8%). Demand-side instruments accounted for the largest share of pairs for population prevention and health promotion (44.3%) and rehabilitation and continuity of care (39.1%), and for 35.7% of pairs associated with risk-factor control and high-risk screening.

### Integration across health-system domains

Medical quality and safety was the most frequent policy-integration domain, occurring in 736 meaning units (42.3%), followed by specialty and stroke-center capacity in 661 (38.0%), chronic disease governance in 411 (23.6%), public health and population prevention in 381 (21.9%), emergency care in 373 (21.4%), rehabilitation and continuity in 281 (16.2%), hierarchical care and referral in 177 (10.2%), and health financing and payment in 135 (7.8%) (Appendix Figure A4; Appendix Table A4).

The stage comparison differed between meaning-unit prevalence and policy coverage. In S1, medical quality and safety occurred in 76 of 150 units (50.7%) and five of six policies. Emergency care occurred in 48 units (32.0%), and public health and population prevention in 40 (26.7%). In S2, public health and population prevention had the highest unit prevalence (126/378, 33.3%), followed by emergency care (117/378, 31.0%), medical quality and safety (107/378, 28.3%), and specialty and stroke-center capacity (103/378, 27.2%). Hierarchical care and referral, specialty and stroke-center capacity, public health and population prevention, and medical quality and safety were each represented in all 13 S2 policies.

In S3, medical quality and safety occurred in 553 of 1,211 units (45.7%) and specialty and stroke-center capacity in 540 (44.6%); both were represented in all 14 policies. Chronic disease governance increased from 3 units in S1 (2.0%) to 83 in S2 (22.0%) and 325 in S3 (26.8%). Rehabilitation and continuity was represented in 11 of 14 S3 policies, and public health and population prevention in 13. Health financing and payment remained less frequent at the unit level, accounting for 9.3% of S1 units, 11.1% of S2 units, and 6.5% of S3 units, but appeared in 4/6, 10/13, and 10/14 policies, respectively.

### Responsible actors and task allocation

Hospitals, medical institutions, and stroke centers were the most frequently specified strict actors, occurring in 295 meaning units (17.0%) and 29 policies. Patients, residents, and high-risk persons assigned an active role occurred in 273 units (15.7%) and 14 policies. Quality-control, technical, and expert bodies occurred in 149 units (8.6%), local health authorities in 108 (6.2%), primary healthcare and public-health institutions in 84 (4.8%), central administrative authorities in 69 (4.0%), territorial or local governments in 64 (3.7%), emergency medical providers in 63 (3.6%), other governmental departments and supporting organizations in 50 (2.9%), and rehabilitation providers in 18 (1.0%).

**Figure 5:**
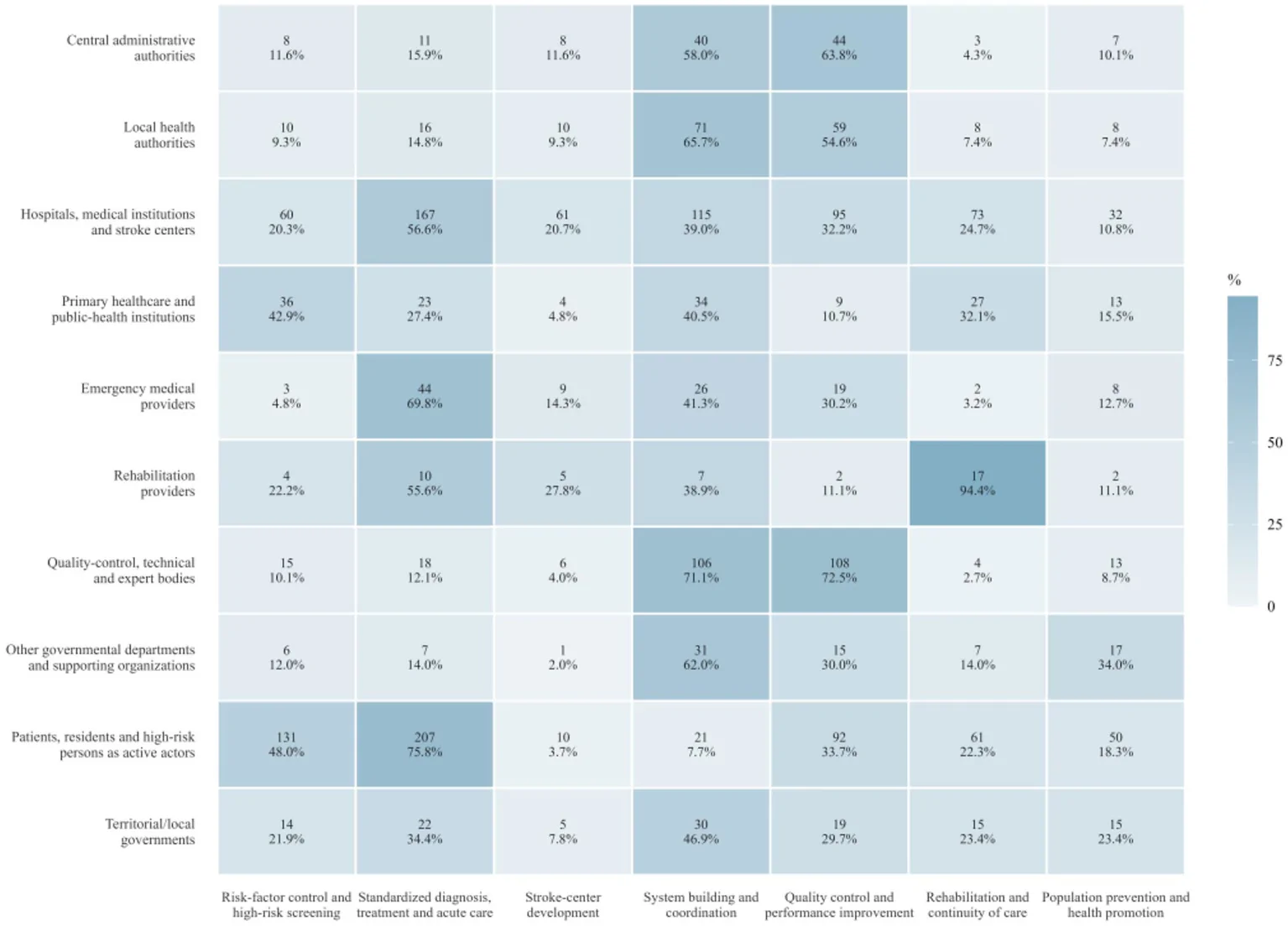
Alignment between explicitly assigned responsible actors and governance tasks. Cells report the number and percentage of meaning units assigned to each strict actor that also contained the corresponding governance task. Strict actors were explicitly named as action-bearing subjects in the current meaning unit. Percentages use the total number of meaning units assigned to the corresponding strict actor as the row-specific denominator. Because a meaning unit could contain multiple governance tasks, percentages across a row do not sum to 100%.

The strict actor-task heatmap showed distinct responsibility profiles (Figure 5). Among units assigned to hospitals, medical institutions, and stroke centers, 56.6% addressed standardized diagnosis, treatment, and acute care, 39.0% system building and coordination, 32.2% quality control and performance improvement, and 24.7% rehabilitation and continuity of care. Among units assigning an active role to patients, residents, or high-risk persons, 75.8% addressed standardized care or acute treatment, 48.0% risk-factor control and screening, 33.7% quality improvement, and 22.3% rehabilitation or continuity of care.

Quality-control, technical, and expert bodies were concentrated in quality control and performance improvement (72.5%) and system building and coordination (71.1%). Local health authorities were most often linked to system building and coordination (65.7%) and quality control and performance improvement (54.6%). Emergency medical providers were predominantly linked to standardized diagnosis, treatment, and acute care (69.8%), whereas rehabilitation providers were strongly concentrated in rehabilitation and continuity of care (94.4%). Percentages used the total number of units assigned to each strict actor as the denominator; because units could contain multiple tasks, percentages within an actor were not mutually exclusive.

### Inter-coder agreement and adjudication

The independently coded sample comprised 348 meaning units, representing 20.0% of the corpus and covering all 33 policies. Exact-set agreement was 14.4% for policy instruments, 44.3% for governance tasks, 63.5% for strict actors, 73.9% for contextual actors, 40.2% for policy objects, and 44.5% for integration domains. Corresponding micro-F1 values were 0.690, 0.817, 0.712, 0.745, 0.786, and 0.790. Cohen’s kappa values across flattened unit-by-code decisions were 0.595 (95% CI 0.557–0.622), 0.746 (0.680–0.787), 0.690 (0.602–0.756), 0.728 (0.634–0.851), 0.719 (0.656–0.758), and 0.720 (0.667–0.757), respectively.

For document functions, the two coders agreed on the primary function for 31 of 33 policies (93.9%), with a Cohen’s kappa of 0.922 (95% CI 0.795–1.000). Exact agreement for the complete function set was also 93.9%, and the micro-F1 was 0.970. Among the 845 independently reviewed Extended-sample units, exact agreement for the complete relevance profile was 63.1% (95% CI 50.9–72.9%), and the micro-F1 across the four relevance criteria was 0.859 (95% CI 0.813–0.899). A separate inclusion-versus-exclusion kappa was not estimable because the reviewed set contained retained units only and both coders classified every record as included.

Adjudication changed at least one of the six meaning-unit dimensions in 66 of the 348 independently coded units and changed the Extended relevance profile in 177 units. The two document-function disagreements were also resolved. All disagreements were adjudicated before analysis, yielding a single consensus dataset containing 33 policies, 1,739 meaning units, and 16,882 assignments. Stage-specific document-function distributions and operational definitions and frequencies of actor categories are reported in Appendix Tables A5, A6, respectively.

## Discussion

### Principal findings

This study examined the national policy design of stroke prevention and stroke center development in China through a multidimensional policy text analysis (25, 27). The findings show that this policy field has evolved from a disease-specific and screening-oriented program into a more system-embedded and quality-oriented governance configuration (6, 7, 15). The final corpus captured a two-layer structure composed of direct stroke policies and broader supporting health-system policies (8, 10, 13). Environment-side instruments remained the dominant steering mechanism, while supply-side instruments supported institutional platforms, capacity building, technical support, and data infrastructure (22, 26). Demand-side instruments were less prominent overall but became more visible in screening, health promotion, service extension, and continuity-oriented arrangements (28, 29). The governance-task analysis further showed that national stroke policy was not organized around a single instrument formula (30). Standardized diagnosis and treatment, quality improvement, system coordination, risk-factor control, rehabilitation continuity, stroke center development, and population prevention were supported by different combinations of instruments (8, 31, 32). Actor analysis also revealed a layered and task-differentiated responsibility structure involving administrative authorities, hospitals and stroke centers, quality-control and technical bodies, primary healthcare and public health institutions, emergency and rehabilitation providers, and patients or residents as active participants (33, 34).

### From disease-specific control to system-embedded governance

A central finding is the shift from disease-specific program formation to system-level embedding (6, 20). In Stage 1, national policy attention was concentrated on screening, high-risk population identification, prevention-base development, early standardization, and foundational quality arrangements (10). These policies established the initial organizational and technical basis for national stroke prevention and control (7). From Stage 2 onward, the number and scope of broader supporting policies increased substantially. Stroke governance became more closely linked with chronic disease prevention, emergency care, hierarchical diagnosis and treatment, rehabilitation, specialty capacity building, county-hospital strengthening, and medical quality improvement (13, 34–36). Comparable policy-text research in another Chinese sector has also identified a progression from foundation-laying and standard setting to implementation-deepening and more refined governance, suggesting that sectoral policy systems often mature through cumulative layering rather than the replacement of earlier arrangements (37).

This two-layer policy structure is important for interpreting the governance logic of the field (30). The main sample carried direct disease-specific policy deployment, while the extended sample provided system-level support and institutional context (7, 8, 38). These two layers worked together to connect stroke-specific tasks with broader national health-system priorities (35, 39). This finding suggests that the governance of stroke in China cannot be fully understood by examining stroke-specific policies alone (20). Broader policies on chronic disease control, emergency care, service delivery, rehabilitation, specialty capacity, payment, and quality governance also shaped the conditions under which stroke prevention and stroke center development were organized (13, 15, 35, 38). In this sense, stroke governance became a policy field located at the intersection of disease control, clinical service capacity, public health, and quality-oriented health-system reform (29, 40).

### Policy instrument mix and task-specific design

The predominance of environment-side instruments suggests that national policy relied heavily on planning, standards, organization, coordination, supervision, and quality monitoring (26, 41). This is understandable in a policy area that requires coordination across prevention, emergency response, hospital treatment, rehabilitation, follow-up, and quality improvement (9, 42). Environment-side instruments provided the institutional rules and steering arrangements needed to define priorities, standardize practice, organize implementation, and monitor performance (15, 33). At the same time, supply-side instruments remained substantial, especially in relation to institution and platform building, capacity building and training, technical support, and data systems (8, 35). These instruments indicate that national stroke policy did not rely only on regulation or planning (43). It also sought to build service capacity and implementation infrastructure (34).

The smaller share of demand-side instruments is also notable (44). Demand-side instruments appeared mainly in screening, early intervention, public education, service extension, continuity of care, and patient participation (28, 32). Their share increased after Stage 1 but remained below that of environment-side and supply-side instruments. This pattern suggests that national policy design continued to place greater emphasis on institutional coordination and service-capacity arrangements than on sustained patient-facing activation or community-based continuity (45, 46). Given that stroke prevention and long-term management depend on risk-factor control, secondary prevention, rehabilitation adherence, follow-up, and self-management, this finding points to an area in which future policy design could be strengthened (47–49).

The instrument-task analysis adds further depth to this interpretation (30). Standardized diagnosis and treatment and stroke center development were supported by both environment-side and supply-side instruments, showing that these tasks required standards and coordination together with institutional platforms and capacity development (8, 31). System building and quality improvement were more clearly environment-oriented, reflecting their reliance on planning, standards, supervision, and monitoring (15, 33). Risk-factor control and screening involved a broader mix of supply-, environment-, and demand-side instruments (32). Rehabilitation and continuity of care drew more strongly on demand-side and supply-side arrangements, while population prevention and health promotion showed the greatest demand-side contribution (24, 28). These patterns indicate that the policy mix was task-specific (30). The relevant question is therefore whether different governance tasks were supported by suitable and mutually reinforcing combinations of tools (43, 50).

### Actor configuration and responsibility allocation

The actor-task analysis further clarifies how policy design translated governance tasks into responsibility structures (45). Under the strict coding rule, hospitals, medical institutions, and stroke centers were the most frequently specified organizational actors, while patients, residents, and high-risk persons were also frequently assigned active roles in screening, treatment participation, self-management, or adherence. Local health authorities, quality-control and technical bodies, primary healthcare and public health institutions, emergency medical providers, and rehabilitation providers occupied more task-specific positions. Contextual-actor coding additionally highlighted the steering role of central administrative authorities. Together, these findings indicate a layered governance structure in which national direction, territorial coordination, hospital-based implementation, technical oversight, frontline service delivery, and patient participation were distributed across different actor groups (15, 36, 39, 51, 52). Cross-sector policy-network evidence from China has likewise shown that stronger coordination, larger core components, and lower fragmentation can support policy coherence and sectoral performance, reinforcing the importance of clearly defined core actors and stable coordination mechanisms (53).

This configuration highlights the central position of hospitals and stroke-related institutional platforms in national policy design (8). Stroke centers were not merely clinical units (54). They served as organizational carriers through which technical standards, workflow optimization, multidisciplinary collaboration, quality monitoring, data reporting, and regional coordination could be connected (8, 21). This helps explain why stroke center development aligned with environment-side and supply-side instruments and why hospitals appeared across standardized care, system building, quality improvement, screening, and rehabilitation-related tasks (54). The stroke center acted as a practical interface between disease-specific policy objectives and wider service-system strengthening (12).

The actor profiles were nevertheless uneven across the continuum of care. Emergency medical providers were concentrated in standardized diagnosis, treatment, and acute-care tasks, while rehabilitation providers were primarily associated with rehabilitation and continuity of care. Patients, residents, and high-risk persons were explicitly represented as active actors, but their responsibilities were concentrated in a narrower set of participation, screening, treatment, and self-management provisions. Primary healthcare and public health institutions also appeared less often than hospitals. This pattern should not be interpreted as evidence that these actors are unimportant in practice (55). It indicates that national policy texts assigned different levels of specificity and breadth to different actors. Because stroke governance depends on prehospital response, acute treatment, rehabilitation, secondary prevention, and long-term self-management (9, 42), clearer cross-setting responsibilities and coordination arrangements may still be needed (52).

### Policy evolution and public health performance

The direction identified in the policy corpus is broadly consistent with, but cannot be assumed to have caused, changes reported in China’s stroke burden and service indicators. Recent national evidence indicates that age-standardized stroke incidence and mortality have declined over the past decade, while prevalence and the absolute burden remain high because of population aging and improved survival (20, 56). At the service level, stroke-center certification in primary hospitals in South China was associated with increased thrombolysis, and implementation of an acute stroke care-map program reduced in-hospital delay (18, 21). These observations are compatible with the stronger standardization, capacity building, and quality governance identified in the later policy stages.

Important gaps nevertheless persist. Multicenter evidence indicates that prehospital delay remains common, and emergency medical service use for acute ischemic stroke remains limited (16, 17). These continuing deficiencies may be consistent with the relatively modest textual prominence of demand-side instruments and the narrower specification of emergency, primary-care, rehabilitation, and patient-facing responsibilities. The present analysis therefore supports a cautious interpretation: system-embedded governance has developed alongside selected improvements in service organization, but policy-text trends alone cannot establish causal effects on incidence, mortality, treatment delay, disability, or other population outcomes.

### Policy implications

Several implications follow from these findings. First, future policy design may benefit from clearer articulation between the direct stroke-policy layer and the supporting health-system-policy layer. The extended policy environment has become increasingly important, but stronger connections may be needed between stroke-specific goals and broader arrangements for chronic disease governance, emergency care, rehabilitation, hierarchical diagnosis and treatment, specialty capacity, financing, and quality improvement (20, 42). Second, demand-side and continuity-oriented instruments could be translated into two operational mechanisms. National quality standards could require stroke centers to establish closed-loop post-discharge pathways specifying electronic referral, rehabilitation linkage, risk-factor monitoring, medication-adherence review, and primary-care follow-up indicators. In parallel, payment and performance arrangements could reward verified completion of secondary-prevention follow-up, community rehabilitation, and structured patient education, with responsibilities allocated across stroke centers, rehabilitation providers, primary-care teams, and patients (47–49, 56). Third, actor responsibilities across the continuum of care could be specified more clearly through defined referral relationships, information-sharing requirements, and evaluation indicators for emergency care, rehabilitation, primary care, quality-control bodies, and patient-facing actors (9, 42, 52). Fourth, stroke centers could be further developed as regional governance platforms coordinating emergency systems, rehabilitation providers, primary care, quality-control bodies, payment mechanisms, and data-reporting systems (8, 12, 56).

### International comparison

The Chinese trajectory shares core features with US and European stroke-system frameworks, including regionalized networks, stroke-center designation, coordinated prehospital and interfacility pathways, and quality monitoring. Recent US scholarship has emphasized definability, accountability, and equity in system performance, whereas the European action plan more explicitly extends system design to organized rehabilitation, follow-up, and life after stroke (54, 57, 58). China’s distinctive feature is the strong embedding of stroke-specific programs within national planning, administrative deployment, Healthy China strategies, county-hospital capacity building, and national quality-control structures. This comparison suggests that China’s system-building strengths could be complemented by more explicit patient-centered continuity standards, cross-institutional data exchange, and accountable post-acute pathways.

### Strengths and limitations

This study has several strengths. It used a source-verified national policy corpus that distinguished stroke-specific policies from broader supporting health-system policies, allowing the analysis to capture both direct policy deployment and system-level embedding. It also applied a multidimensional policy design framework linking document function, policy instruments, governance tasks, strict and contextual actors, policy objects, and policy integration. Atomic meaning-unit segmentation reduced inflation from complex clauses, while the final codebook made the boundaries between actors, policy objects, instruments, and integration domains explicit. In addition, a stratified 20% sample of meaning units was independently reviewed across all six dimensions, all document functions and Extended relevance records were reviewed, and disagreements were resolved through documented consensus adjudication. These procedures strengthened the traceability and internal consistency of the final corpus.

Several limitations should also be acknowledged. First, the study focused on national policy texts and did not examine provincial or local policy implementation. It therefore cannot assess regional variation in policy translation, resource allocation, actor coordination, or execution. This is important because recent Chinese policy research in another sector has shown that regional development trajectories may be associated with distinct intervention logics and policy mixes (59). Second, the analysis addressed policy design rather than implementation outcomes, so it cannot determine whether the observed configurations improved service delivery, quality of care, disability reduction, or population health. Third, the Extended sample was restricted to provisions with a clearly traceable relationship to stroke governance. It should therefore be interpreted as representing the broader policy environment directly relevant to stroke prevention and care rather than every general health-system reform with only indirect implications for stroke services. Fourth, the selection of Extended clauses and assignment of integration domains still involved interpretive judgment, although predefined eligibility rules, source verification, independent review, and consensus adjudication were used to reduce this risk. Fifth, strict actor coding intentionally required explicit identification within the current meaning unit. Although contextual-actor coding was reported separately, actors that remained implicit may still have been underestimated. Sixth, frequency and prevalence describe textual emphasis and coverage, not the substantive importance or effectiveness of a policy measure. Finally, policy texts provide only one view of governance. Future research could link national policy design with provincial and local implementation documents, policy-network data, service-quality indicators, stroke-center performance, financing arrangements, and patient-level outcomes to examine how these configurations operate in practice.

## Data Availability

The original contributions presented in the study are included in the article/Supplementary material, further inquiries can be directed to the corresponding author.
