# Supplementary material for: From disease-specific control to system-embedded governance: a multidimensional policy design analysis of stroke prevention and stroke center development in China
Source: Front Public Health. 2026 Aug 10;14:1888583. doi: 10.3389/fpubh.2026.1888583 (PMC13500510; doi:10.3389/fpubh.2026.1888583)
Supplement: Supplementary file 1 [file Data_Sheet_1.docx]

**Appendix Figure A1. Distribution and policy coverage of detailed policy instruments.**

Note. Bars show the percentage of the 1,739 meaning units assigned to each of the 14 detailed policy-instrument codes. Labels report the corresponding number of meaning units and the number of policies containing at least one assignment of that code. Because meaning units could receive multiple policy-instrument codes, percentages across codes do not sum to 100%.

**Appendix Figure A2. Detailed policy-instrument prevalence in the Main and Extended samples.**

Note. Points show the percentage of meaning units within each sample assigned to each detailed policy-instrument code. The denominators were 894 Main-sample meaning units and 845 Extended-sample meaning units. Codes were non-exclusive, so percentages within a sample do not sum to 100%.

**Appendix Figure A3. Stage-specific prevalence of governance tasks.**

Note. Cells show the number and percentage of meaning units within each stage assigned to each governance task. Stage-specific denominators were 150 meaning units in S1, 378 in S2, and 1,211 in S3. A meaning unit could receive more than one governance-task code; therefore, percentages across tasks are not mutually exclusive.

**Appendix Figure A4. Policy integration across stages.**

Note. Panel A shows the prevalence of each integration domain among meaning units within each stage. Panel B shows policy coverage, defined as the number and percentage of policies in each stage containing at least one meaning unit assigned to the corresponding integration domain. Cell labels report counts and percentages. Stage-specific denominators were 150 meaning units and 6 policies in S1, 378 meaning units and 13 policies in S2, and 1,211 meaning units and 14 policies in S3. Integration domains were non-exclusive.
