# Supplementary material for: From disease-specific control to system-embedded governance: a multidimensional policy design analysis of stroke prevention and stroke center development in China
Source: Front Public Health. 2026 Aug 10;14:1888583. doi: 10.3389/fpubh.2026.1888583 (PMC13500510; doi:10.3389/fpubh.2026.1888583)
Supplement: Supplementary file 2 [file Data_Sheet_2.docx]

**Appendix Table A1. Full titles, issuing authorities, and official sources for the included national policies.**

| **ID** | **Year** | **Original Chinese title** | **English translation** | **Issuing authority** | **Sample/stage** | **Official source** | **Official source URL** |
| --- | --- | --- | --- | --- | --- | --- | --- |
| P14 | 2009 | 医疗质量控制中心管理办法（试行） | Measures for the Administration of Medical Quality Control Centers (Trial) | Ministry of Health | Ext./S1 | NHC | https://www.nhc.gov.cn/bgt/s9510/200906/af0b2f1dcde649f48e5b670c8f0e3b82.shtml |
| P02 | 2011 | 关于做好脑卒中规范诊疗和医疗质量控制工作的通知 | Notice on Strengthening Standardized Stroke Diagnosis and Treatment and Medical Quality Control | General Office, Ministry of Health | Main/S1 | NHC | https://www.nhc.gov.cn/zwgkzt/pyzgl1/201206/55131.shtml |
| P03 | 2012 | 卫生部办公厅关于印发《脑卒中高危人群筛查和干预试点项目管理办法（试行）》的通知 | Notice on Issuing the Administrative Measures for the Pilot Program of Screening and Intervention for Populations at High Risk of Stroke (Trial) | General Office, Ministry of Health | Main/S1 | NHC | https://www.nhc.gov.cn/wjw/gfxwj/201204/c0b06b0816e64159bafbe234271a7fd6.shtml |
| P04 | 2012 | 卫生部办公厅关于同意首都医科大学宣武医院等47家医院作为卫生部脑卒中筛查与防治基地的通知 | Notice Approving Xuanwu Hospital of Capital Medical University and 46 Other Hospitals as Ministry of Health Stroke Screening and Prevention Bases | General Office, Ministry of Health | Main/S1 | NHC | https://www.nhc.gov.cn/zwgkzt/pyzgl1/201206/55132.shtml |
| N01 | 2013 | 关于印发脑卒中等8个常见病种（手术）康复医疗双向转诊标准（试行）的通知 | Notice on Issuing the Bidirectional Referral Standards for Rehabilitation Care for Stroke and Seven Other Common Diseases (Procedures) (Trial) | National Health and Family Planning Commission | Ext./S1 | NHC | https://www.nhc.gov.cn/yzygj/c100068/201304/87ef190231674aaaa56f6cf35a259b4c.shtml |
| P17 | 2013 | 院前医疗急救管理办法 | Measures for the Administration of Prehospital Emergency Medical Care | National Health and Family Planning Commission | Ext./S1 | NHC | https://www.nhc.gov.cn/fzs/c100048/201808/6878dddd2a3d48c59de1c43a6c68423d.shtml |
| P06 | 2015 | 国家卫生计生委办公厅关于提升急性心脑血管疾病医疗救治能力的通知 | Notice on Improving Medical Treatment Capacity for Acute Cardiovascular and Cerebrovascular Diseases | General Office, National Health and Family Planning Commission | Ext./S2 | NHC | https://www.nhc.gov.cn/yzygj/c100068/201503/142a8815094147de97acef0eb34a2e51.shtml |
| P10 | 2015 | 国务院办公厅关于推进分级诊疗制度建设的指导意见 | Guiding Opinions on Advancing the Development of a Tiered Diagnosis and Treatment System | General Office of the State Council | Ext./S2 | NHC | https://www.nhc.gov.cn/yzygj/c100068/201509/1d374d8faef1403b8d0d41a840ff4e73.shtml |
| P07 | 2016 | 国家卫生计生委办公厅关于印发医院卒中中心建设与管理指导原则（试行）的通知 | Notice on Issuing the Guiding Principles for the Development and Management of Hospital Stroke Centers (Trial) | General Office, National Health and Family Planning Commission | Main/S2 | NHC | https://www.nhc.gov.cn/zwgk/jdjd/201611/c917760206cd4c13b06c9cb812cd6a2a.shtml |
| P08 | 2016 | 脑卒中综合防治工作方案 | Comprehensive Plan for Stroke Prevention and Control | General Office, National Health and Family Planning Commission; Office of the National Administration of Traditional Chinese Medicine | Main/S2 | NHC | https://www.nhc.gov.cn/jkj/c100062/201612/43978c7df4324ba8a13a8c1507069b89.shtml |
| P09 | 2016 | “健康中国2030”规划纲要 | Healthy China 2030 Planning Outline | CPC Central Committee and State Council | Ext./S2 | NHC | https://www.nhc.gov.cn/guihuaxxs/c100132/201610/cef9821abcfc4544bb27e2bc533bd7cf.shtml |
| P11 | 2016 | 国务院关于印发“十三五”卫生与健康规划的通知 | 13th Five-Year Plan for Health | State Council | Ext./S2 | NHC | https://www.nhc.gov.cn/bgt/gwywj2/201701/6de27746052c4d119f83482410ffa932.shtml |
| P12 | 2017 | 中国防治慢性病中长期规划（2017—2025年） | China's Medium- and Long-Term Plan for the Prevention and Treatment of Chronic Diseases (2017-2025) | General Office of the State Council | Ext./S2 | NDRC | https://www.ndrc.gov.cn/fggz/fzzlgh/gjjzxgh/201705/t20170511_1196751.html |
| P13 | 2017 | 进一步改善医疗服务行动计划（2018—2020年） | Action Plan for Further Improving Medical Services (2018-2020) | National Health and Family Planning Commission; National Administration of Traditional Chinese Medicine | Ext./S2 | NHC | https://www.nhc.gov.cn/yzygj/c100068/201801/0aa6971b338e4a71a9be96a719567580.shtml |
| P18 | 2018 | 关于进一步加强脑卒中诊疗管理相关工作的通知 | Notice on Further Strengthening the Management of Stroke Diagnosis and Treatment | General Office, National Health and Family Planning Commission | Main/S2 | NHC | http://www.nhc.gov.cn/yzygj/s7659/201804/d79dfff31feb4ab7960f827a5ea27d26.shtml |
| N02 | 2019 | 脑卒中人群筛查及综合干预技术方案（国卫办疾控函〔2019〕577号附件2） | Technical Plan for Stroke Population Screening and Comprehensive Intervention | General Office, National Health Commission | Main/S2 | NHC | https://www.nhc.gov.cn/jkj/c100063/201907/aebd8901fe284b6bacb45da8dfa1a0c7/files/1733138021409_89647.pdf |
| P15 | 2019 | 国务院关于实施健康中国行动的意见 | Opinions of the State Council on Implementing the Healthy China Initiative | State Council | Ext./S2 | NHC | https://www.nhc.gov.cn/guihuaxxs/jkzcwj/201907/e5720cde669347c48cf82f050c253f9c.shtml |
| P16 | 2019 | 健康中国行动（2019—2030年） | Healthy China Initiative (2019-2030) | Healthy China Initiative Promotion Committee | Ext./S2 | NHC | https://www.nhc.gov.cn/guihuaxxs/c100133/201907/2a6ed52f1c264203b5351bdbbadd2da8.shtml |
| N03 | 2020 | 关于印发进一步完善院前医疗急救服务指导意见的通知 | Guiding Opinions on Further Improving Prehospital Emergency Medical Services | National Health Commission and other departments | Ext./S2 | NHC | https://www.nhc.gov.cn/wjw/c100175/202009/07e1ddf9a8ee40b5a45dc8c89b5cd3c3.shtml |
| N04 | 2021 | 关于印发加快推进康复医疗工作发展意见的通知 | Opinions on Accelerating the Development of Rehabilitation Medical Services | National Health Commission and other departments | Ext./S3 | NHC | https://www.nhc.gov.cn/yzygj/c100068/202106/54832e92612a4a2c947b8a36d7c305ca.shtml |
| P19 | 2021 | 关于印发加强脑卒中防治工作减少百万新发残疾工程综合方案的通知 | Comprehensive Plan for Strengthening Stroke Prevention and Control and Reducing One Million New Disabilities | National Health Commission and nine other departments | Main/S3 | NHC | https://www.nhc.gov.cn/wjw/c100175/202106/2b05b16362d344d189af5bc6a3cc72e1.shtml |
| P20 | 2021 | 国家卫生健康委办公厅关于印发《中国脑卒中防治指导规范（2021年版）》的通知 | Chinese Guidelines for Stroke Prevention and Treatment (2021 Edition) | General Office, National Health Commission | Main/S3 | NHC | https://www.nhc.gov.cn/yzygj/c100068/202108/682b5a958a7443b2b8269ab9303cfbeb.shtml |
| P21 | 2021 | 中华人民共和国国民经济和社会发展第十四个五年规划和2035年远景目标纲要 | Outline of the 14th Five-Year Plan for National Economic and Social Development and the Long-Range Objectives Through 2035 | National People's Congress | Ext./S3 | NDRC | https://www.ndrc.gov.cn/xxgk/zcfb/ghwb/202103/t20210323_1270124.html |
| P23 | 2021 | “十四五”国家临床专科能力建设规划 | 14th Five-Year National Plan for Clinical Specialty Capacity Building | National Health Commission | Ext./S3 | NHC | https://www.nhc.gov.cn/yzygj/c100068/202110/eea7c34917704226a1589b97cdf0a516.shtml |
| P24 | 2021 | “千县工程”县医院综合能力提升工作方案（2021—2025年） | Thousand-County Project Work Plan for Enhancing the Comprehensive Capacity of County Hospitals (2021-2025) | General Office, National Health Commission | Ext./S3 | NHC | https://www.nhc.gov.cn/yzygj/c100068/202111/1cd70df7b2474daeb76917759a2e2949.shtml |
| P22 | 2022 | “十四五”国民健康规划 | 14th Five-Year Plan for National Health | General Office of the State Council | Ext./S3 | NDRC | https://www.ndrc.gov.cn/fggz/fzzlgh/gjjzxgh/202206/t20220601_1326725.html |
| N05 | 2023 | 医疗质量控制中心管理规定 | Provisions on the Administration of Medical Quality Control Centers | General Office, National Health Commission | Ext./S3 | NHC | https://www.nhc.gov.cn/yzygj/c100068/202302/9718af196a444f95b37afb73e9047305.shtml |
| P25 | 2023 | 国家卫生健康委办公厅关于成立国家卫生健康委加强脑卒中防治工作减少百万新发残疾工程专家委员会的通知 | Notice on Establishing the Expert Committee for the Project to Strengthen Stroke Prevention and Control and Reduce One Million New Disabilities | General Office, National Health Commission | Main/S3 | NHC | https://www.nhc.gov.cn/yzygj/c100068/202305/10a2fe1288ca47f589078f9888e4442a.shtml |
| P26 | 2023 | 健康中国行动—心脑血管疾病防治行动实施方案（2023—2030年） | Healthy China Initiative: Action Plan for the Prevention and Control of Cardiovascular and Cerebrovascular Diseases (2023-2030) | National Health Commission and 13 other departments | Ext./S3 | NHC | https://www.nhc.gov.cn/ylyjs/gzdt/202311/a40dcf8a65314b818c46c9d1e683b9c3.shtml |
| N06 | 2024 | 国家卫生健康委办公厅关于确定省级心血管病和脑血管病防治技术牵头单位的通知 | Notice on Designating Provincial Lead Technical Institutions for the Prevention and Control of Cardiovascular and Cerebrovascular Diseases | General Office, National Health Commission | Ext./S3 | NHC | https://www.nhc.gov.cn/ylyjs/gzdt/202404/a9da29dc36424652a456b7ea1d12a8dc.shtml |
| N07 | 2024 | 国家卫生健康委办公厅关于印发《脑血管病防治指南（2024年版）》的通知及附件 | Guidelines for the Prevention and Control of Cerebrovascular Diseases (2024 Edition) | General Office, National Health Commission | Main/S3 | NHC | https://www.nhc.gov.cn/ylyjs/zcwj/202412/ba037e931fff4870930f65ff667ea9ed.shtml |
| P27 | 2026 | 国务院办公厅印发《关于加快建设分级诊疗体系的若干措施》的通知 | Several Measures to Accelerate the Development of a Tiered Diagnosis and Treatment System | General Office of the State Council | Ext./S3 | NHC | https://www.nhc.gov.cn/wjw/mtbd/202604/7f64a7d2f54c4f7197692ff18869b02a.shtml |
| P28 | 2026 | 国家卫生健康委办公厅关于印发2026年国家医疗质量安全改进目标的通知（含各专业质控工作改进目标附件） | 2026 National Medical Quality and Safety Improvement Targets | General Office, National Health Commission | Ext./S3 | NHC | https://www.nhc.gov.cn/yzygj/c100068/202603/9f642951b99c447f8cff9da8abb74dc3.shtml |

**** English titles are author translations of the official Chinese titles. Ext. denotes the Extended sample. S1, S2, and S3 denote 2009-2014, 2015-2020, and 2021-2026, respectively. Main policies directly addressed stroke-specific prevention, care, rehabilitation, stroke-center development, or quality governance; Extended policies contributed only provisions with an explicit or clearly traceable relationship to the stroke pathway or its enabling health-system environment. Official source URLs link to the government webpages or official attachments used for the analysis.

**Appendix Table A2. Detailed policy-instrument frequencies and policy coverage overall and by stage.**

| **Code** | **Detailed policy instrument** | **Overall units, n (%)** | **Overall policies, n (%)** | **S1 units, n (%)** | **S1 policies, n (%)** | **S2 units, n (%)** | **S2 policies, n (%)** | **S3 units, n (%)** | **S3 policies, n (%)** |
| --- | --- | --- | --- | --- | --- | --- | --- | --- | --- |
| PI-S1 | Institution/platform/network building | 484 (27.8) | 32 (97.0) | 20 (13.3) | 5 (83.3) | 85 (22.5) | 13 (100.0) | 379 (31.3) | 14 (100.0) |
| PI-S2 | Workforce and capacity building | 276 (15.9) | 32 (97.0) | 14 (9.3) | 6 (100.0) | 62 (16.4) | 12 (92.3) | 200 (16.5) | 14 (100.0) |
| PI-S3 | Technical and professional support | 456 (26.2) | 30 (90.9) | 13 (8.7) | 4 (66.7) | 49 (13.0) | 12 (92.3) | 394 (32.5) | 14 (100.0) |
| PI-S4 | Data, registry and information systems | 332 (19.1) | 32 (97.0) | 28 (18.7) | 5 (83.3) | 69 (18.3) | 13 (100.0) | 235 (19.4) | 14 (100.0) |
| PI-S5 | Direct resource and financial input | 149 (8.6) | 26 (78.8) | 10 (6.7) | 4 (66.7) | 32 (8.5) | 10 (76.9) | 107 (8.8) | 12 (85.7) |
| PI-E1 | Strategic planning and target setting | 341 (19.6) | 32 (97.0) | 13 (8.7) | 5 (83.3) | 45 (11.9) | 13 (100.0) | 283 (23.4) | 14 (100.0) |
| PI-E2 | Standards, norms and procedures | 763 (43.9) | 33 (100.0) | 50 (33.3) | 6 (100.0) | 114 (30.2) | 13 (100.0) | 599 (49.5) | 14 (100.0) |
| PI-E3 | Supervision, accountability and compliance | 368 (21.2) | 25 (75.8) | 27 (18.0) | 4 (66.7) | 27 (7.1) | 9 (69.2) | 314 (25.9) | 12 (85.7) |
| PI-E4 | Organization, coordination and responsibility allocation | 428 (24.6) | 33 (100.0) | 39 (26.0) | 6 (100.0) | 121 (32.0) | 13 (100.0) | 268 (22.1) | 14 (100.0) |
| PI-E5 | Quality monitoring, evaluation and improvement | 469 (27.0) | 32 (97.0) | 22 (14.7) | 5 (83.3) | 69 (18.3) | 13 (100.0) | 378 (31.2) | 14 (100.0) |
| PI-E6 | Pricing, payment and incentive arrangements | 107 (6.2) | 19 (57.6) | 5 (3.3) | 2 (33.3) | 26 (6.9) | 8 (61.5) | 76 (6.3) | 9 (64.3) |
| PI-D1 | Screening, risk identification and early intervention | 484 (27.8) | 25 (75.8) | 22 (14.7) | 3 (50.0) | 51 (13.5) | 10 (76.9) | 411 (33.9) | 12 (85.7) |
| PI-D2 | Public education, participation and self-management support | 257 (14.8) | 25 (75.8) | 5 (3.3) | 2 (33.3) | 53 (14.0) | 12 (92.3) | 199 (16.4) | 11 (78.6) |
| PI-D3 | Service access, referral and continuity | 349 (20.1) | 28 (84.8) | 16 (10.7) | 3 (50.0) | 92 (24.3) | 13 (100.0) | 241 (19.9) | 12 (85.7) |

**** Unit percentages use 1,739 meaning units overall and stage-specific denominators of 150, 378, and 1,211 for S1, S2, and S3, respectively. Policy percentages use 33 policies overall and stage-specific denominators of 6, 13, and 14. Policy-instrument codes were non-exclusive; therefore, percentages across rows do not sum to 100%.

**Appendix Table A3. Instrument-task alignment at the meaning-unit level.**

| **Task code** | **Governance task** | **Task units** | **Task-instrument pair total** | **Supply-side instruments, n (%)** | **Environment-side instruments, n (%)** | **Demand-side instruments, n (%)** |
| --- | --- | --- | --- | --- | --- | --- |
| TK-01 | Risk-factor control and high-risk screening | 479 | 1035 | 283 (27.3) | 383 (37.0) | 369 (35.7) |
| TK-02 | Standardized diagnosis, treatment and acute care | 806 | 1732 | 541 (31.2) | 720 (41.6) | 471 (27.2) |
| TK-03 | Stroke-center development | 87 | 206 | 80 (38.8) | 81 (39.3) | 45 (21.8) |
| TK-04 | System building and coordination | 557 | 1051 | 438 (41.7) | 459 (43.7) | 154 (14.7) |
| TK-05 | Quality control and performance improvement | 590 | 1219 | 447 (36.7) | 557 (45.7) | 215 (17.6) |
| TK-06 | Rehabilitation and continuity of care | 270 | 626 | 175 (28.0) | 206 (32.9) | 245 (39.1) |
| TK-07 | Population prevention and health promotion | 216 | 460 | 121 (26.3) | 135 (29.3) | 204 (44.3) |

**** Each governance-task and major-instrument-category pair was counted no more than once per meaning unit. The task-units column reports the number of meaning units assigned to each governance task. Pair percentages are calculated within each governance task against the total number of task-instrument major-category pairs for that task; therefore, the three major-instrument percentages in each row sum to 100%.

**Appendix Table A4. Policy-integration domains overall and by stage.**

| **Code** | **Integration domain** | **Overall units, n (%)** | **Overall policies, n (%)** | **S1 units, n (%)** | **S1 policies, n (%)** | **S2 units, n (%)** | **S2 policies, n (%)** | **S3 units, n (%)** | **S3 policies, n (%)** |
| --- | --- | --- | --- | --- | --- | --- | --- | --- | --- |
| INT-01 | Chronic disease governance | 411 (23.6) | 22 (66.7) | 3 (2.0) | 1 (16.7) | 83 (22.0) | 11 (84.6) | 325 (26.8) | 10 (71.4) |
| INT-02 | Emergency care | 373 (21.4) | 21 (63.6) | 48 (32.0) | 1 (16.7) | 117 (31.0) | 11 (84.6) | 208 (17.2) | 9 (64.3) |
| INT-03 | Rehabilitation and continuity | 281 (16.2) | 24 (72.7) | 10 (6.7) | 2 (33.3) | 73 (19.3) | 11 (84.6) | 198 (16.4) | 11 (78.6) |
| INT-04 | Hierarchical care and referral | 177 (10.2) | 26 (78.8) | 8 (5.3) | 3 (50.0) | 85 (22.5) | 13 (100.0) | 84 (6.9) | 10 (71.4) |
| INT-05 | Specialty and stroke-center capacity | 661 (38.0) | 31 (93.9) | 18 (12.0) | 4 (66.7) | 103 (27.2) | 13 (100.0) | 540 (44.6) | 14 (100.0) |
| INT-06 | Public health and population prevention | 381 (21.9) | 29 (87.9) | 40 (26.7) | 3 (50.0) | 126 (33.3) | 13 (100.0) | 215 (17.8) | 13 (92.9) |
| INT-07 | Medical quality and safety | 736 (42.3) | 32 (97.0) | 76 (50.7) | 5 (83.3) | 107 (28.3) | 13 (100.0) | 553 (45.7) | 14 (100.0) |
| INT-08 | Health financing and payment | 135 (7.8) | 24 (72.7) | 14 (9.3) | 4 (66.7) | 42 (11.1) | 10 (76.9) | 79 (6.5) | 10 (71.4) |

** Overall unit percentages use 1,739 meaning units and overall policy percentages use 33 policies. Stage-specific unit denominators were 150, 378, and 1,211, and stage-specific policy denominators were 6, 13, and 14 for S1, S2, and S3, respectively. Integration domains were non-exclusive because a meaning unit or policy could be linked to more than one health-system domain.

**Appendix Table A5. Document functions overall and by stage.**

| **Code** | **Document function** | **Overall primary, n** | **Overall secondary, n** | **Overall any function, n (%)** | **S1 any function, n (%)** | **S2 any function, n (%)** | **S3 any function, n (%)** |
| --- | --- | --- | --- | --- | --- | --- | --- |
| DF-01 | Strategic planning | 12 | 0 | 12 (36.4) | 0 (0.0) | 7 (53.8) | 5 (35.7) |
| DF-02 | Administrative deployment | 6 | 8 | 14 (42.4) | 3 (50.0) | 5 (38.5) | 6 (42.9) |
| DF-03 | Technical guidance | 5 | 2 | 7 (21.2) | 2 (33.3) | 3 (23.1) | 2 (14.3) |
| DF-04 | Institutional construction | 4 | 4 | 8 (24.2) | 2 (33.3) | 2 (15.4) | 4 (28.6) |
| DF-05 | Service-system arrangement | 3 | 15 | 18 (54.5) | 2 (33.3) | 8 (61.5) | 8 (57.1) |
| DF-06 | Quality governance and target setting | 3 | 4 | 7 (21.2) | 3 (50.0) | 1 (7.7) | 3 (21.4) |
| DF-07 | Financing and payment support | 0 | 0 | 0 (0.0) | 0 (0.0) | 0 (0.0) | 0 (0.0) |

** Each policy received one primary document-function assignment and could receive one secondary assignment when a clearly separable additional role was present. Any-function counts represent the union of primary and secondary assignments; therefore, categories are non-exclusive at the any-function level. Overall percentages use 33 policies, and stage-specific percentages use 6, 13, and 14 policies in S1, S2, and S3, respectively.

**Appendix Table A6. Operational definitions and frequencies of responsible-actor categories.**

| **Code** | **Actor category** | **Operational definition** | **Strict units, n (%)** | **Strict policies, n (%)** | **Contextual units, n (%)** | **Contextual policies, n (%)** | **Combined units, n (%)** | **Combined policies, n (%)** |
| --- | --- | --- | --- | --- | --- | --- | --- | --- |
| ACT-01 | Central administrative authorities | Central ministries, commissions, State Council bodies or national administrative authorities assigned an implementation, coordination or supervisory role. | 69 (4.0) | 21 (63.6) | 132 (7.6) | 20 (60.6) | 164 (9.4) | 23 (69.7) |
| ACT-02 | Local health authorities | Provincial, municipal, county or territorial health authorities assigned a role. | 108 (6.2) | 23 (69.7) | 104 (6.0) | 22 (66.7) | 138 (7.9) | 24 (72.7) |
| ACT-03 | Hospitals, medical institutions and stroke centers | Hospitals, base hospitals, medical institutions or stroke centers assigned a clinical, managerial, training or coordinating role. | 295 (17.0) | 29 (87.9) | 242 (13.9) | 21 (63.6) | 324 (18.6) | 29 (87.9) |
| ACT-04 | Primary healthcare and public-health institutions | Community health centers, township hospitals, family-doctor teams, CDCs or public-health institutions assigned a role. | 84 (4.8) | 18 (54.5) | 64 (3.7) | 14 (42.4) | 96 (5.5) | 18 (54.5) |
| ACT-05 | Emergency medical providers | Emergency centers, ambulance systems, dispatch centers or emergency departments assigned an action. | 63 (3.6) | 11 (33.3) | 68 (3.9) | 10 (30.3) | 70 (4.0) | 11 (33.3) |
| ACT-06 | Rehabilitation providers | Rehabilitation hospitals, rehabilitation departments, rehabilitation centers or rehabilitation professionals assigned an action. | 18 (1.0) | 9 (27.3) | 16 (0.9) | 8 (24.2) | 18 (1.0) | 9 (27.3) |
| ACT-07 | Quality-control, technical and expert bodies | Quality-control centers, technical lead units, expert committees or professional technical organizations assigned a role. | 149 (8.6) | 18 (54.5) | 53 (3.0) | 12 (36.4) | 154 (8.9) | 18 (54.5) |
| ACT-08 | Other governmental departments and supporting organizations | Non-health government departments, insurers, education bodies, civil-affairs bodies, disability organizations and similar supporting actors. | 50 (2.9) | 16 (48.5) | 116 (6.7) | 12 (36.4) | 129 (7.4) | 16 (48.5) |
| ACT-09 | Patients, residents and high-risk persons as active actors | Patients, residents or high-risk persons explicitly required or encouraged to perform an action such as self-assessment, self-management or adherence. | 273 (15.7) | 14 (42.4) | 174 (10.0) | 8 (24.2) | 273 (15.7) | 14 (42.4) |
| ACT-10 | Territorial/local governments | Local governments or territorial jurisdictions assigned an action when no functional department is identified. | 64 (3.7) | 18 (54.5) | 67 (3.9) | 17 (51.5) | 78 (4.5) | 19 (57.6) |

**** Unit percentages use all 1,739 meaning units, and policy percentages use all 33 policies. Strict actors were explicitly named as action-bearing subjects in the current meaning unit. Contextual actors were inherited only from an explicitly named actor within the same natural paragraph, numbered provision, or immediately adjacent sentence under the predefined contextual rule. Combined counts represent the union of strict and contextual assignments and should not be calculated by adding the two columns.

**Appendix Table A7. Operational coding framework and decision rules.**

**Section: Document functions**

| **Code** | **Operational label** | **Operational definition** | **Inclusion indicators** | **Key exclusion or boundary** |
| --- | --- | --- | --- | --- |
| DF-01 | Strategic planning | Positions a document as a long-range, cross-sector or system-level plan with broad goals and development direction. | National plans, outlines and medium/long-term strategies with explicit system goals. | Single-task implementation notices or technical guidance. Document-level only; may coexist with another function when the document has a clearly separate operational role. |
| DF-02 | Administrative deployment | Mobilizes implementation through formal assignments, deadlines, reporting requirements or administrative action. | Notices, work plans and action arrangements that assign work and require implementation. | Pure technical guidance without administrative deployment. Use when implementation mobilization is a principal document function. |
| DF-03 | Technical guidance | Provides clinical, technical or operational guidance for prevention, diagnosis, treatment, rehabilitation or management. | Guidelines, technical protocols and standardized clinical pathways. | General planning without technical content. A document may also have a service-arrangement function. |
| DF-04 | Institutional construction | Creates, designates or restructures formal institutions, centers, networks or technical lead units. | Stroke centers, quality-control centers, expert committees or lead institutions. | Statements merely encouraging coordination without institutional construction. Document-level role; unit-level construction is coded as PI-S1. |
| DF-05 | Service-system arrangement | Organizes service delivery, referral, emergency, rehabilitation or continuity across settings and levels. | Referral standards, emergency-network arrangements and rehabilitation-service networks. | Technical treatment recommendations without service organization. Use when service organization is a principal function. |
| DF-06 | Quality governance and target setting | Sets quality goals, monitoring systems, indicators, assessment arrangements or quality-control structures. | National quality targets, quality-control-center regulations and performance-improvement documents. | Routine data reporting without evaluative use. Document-level role; unit-level content uses PI-E5 and/or TK-05. |
| DF-07 | Financing and payment support | Uses price, insurance payment, direct public investment or financial-support arrangements as a major document function. | Documents primarily organized around provider payment, service prices or earmarked funding. | A single payment clause in an otherwise unrelated document. If financing is only one supporting clause, retain the main function and code the clause with PI-E6 or PI-S5. |

**Section: Policy instruments—supply-side**

| **Code** | **Operational label** | **Operational definition** | **Inclusion indicators** | **Key exclusion or boundary** |
| --- | --- | --- | --- | --- |
| PI-S1 | Institution/platform/network building | Creates, designates, expands or strengthens an institutional carrier, center, network, system or formal service platform. | Build or designate stroke centers, emergency centers, rehabilitation networks, quality centers or information platforms. | Responsibility allocation without construction; direct service extension without a platform. Co-code PI-E4 only when both construction and coordination/responsibility are explicit. |
| PI-S2 | Workforce and capacity building | Builds personnel competence or service capacity through training, staffing, skill improvement or capability enhancement. | Training, staffing standards, capacity enhancement and competency development. | Technical advice with no training/capacity component. Co-code PI-S3 when technical support and training are separately explicit. |
| PI-S3 | Technical and professional support | Provides expert guidance, technical assistance, professional consultation, tele-support or dissemination of appropriate technologies. | Expert guidance, technical support, teleconsultation and professional assistance. | Training-only provisions. Do not code general standards as technical support unless an assisting actor or support mechanism is present. |
| PI-S4 | Data, registry and information systems | Creates or operates data collection, reporting, registry, electronic record, information-sharing or digital-management systems. | Registries, reporting platforms, electronic health records, information exchange and data extraction. | Monitoring/evaluation without a data-system component. Co-code PI-E5 only when the data are explicitly used for evaluation, feedback or improvement. |
| PI-S5 | Direct resource and financial input | Provides direct public investment, earmarked funding, equipment, facilities or other material resources. | Government investment, special funds, facility/equipment allocation and direct financial support. | Pricing, insurance payment or performance incentives. Use PI-E6 for payment rules and incentive structures; co-code only when both direct input and payment design are explicit. |

**Section: Policy instruments—environment-side**

| **Code** | **Operational label** | **Operational definition** | **Inclusion indicators** | **Key exclusion or boundary** |
| --- | --- | --- | --- | --- |
| PI-E1 | Strategic planning and target setting | Sets formal targets, implementation horizons, quantified goals or stage priorities. | Time-bound targets, quantified coverage and explicit strategic objectives. | General aspirations with no implementable direction or target. A broad statement is codable only when it contains a clear target or implementation direction. |
| PI-E2 | Standards, norms and procedures | Issues or requires standards, criteria, procedures, workflows, clinical norms or institutional rules. | Transfer criteria, technical norms, standard workflows and formal procedures. | General quality goals without a standard/procedure component. Referral criteria are PI-E2; the service linkage created by them may additionally be PI-D3. |
| PI-E3 | Supervision, accountability and compliance | Requires supervision, inspection, accountability, compliance enforcement or administrative assessment. | Inspection, supervision, accountability and corrective requirements. | Quality monitoring used only for feedback and improvement. Co-code PI-E5 when both supervisory pressure and evaluative quality improvement are explicit. |
| PI-E4 | Organization, coordination and responsibility allocation | Allocates responsibilities or explicitly coordinates, integrates or links actors, organizations, levels, services or settings. | Named actor responsibilities, cross-sector collaboration, joint mechanisms, explicit multi-setting linkage, integration or role differentiation. | Institution/network construction without explicit coordination; simple transfer/access language without role or organizational linkage. Use for explicit linkage, integration, joint participation, or division of responsibilities involving at least two actors, settings, or service components; do not infer coordination from a platform name alone. |
| PI-E5 | Quality monitoring, evaluation and improvement | Uses indicators, monitoring, evaluation, feedback or improvement cycles to govern quality or performance. | Quality indicators, performance evaluation, feedback and improvement requirements. | Data collection/reporting alone. Performance assessment linked to personnel incentives may also receive PI-E6. |
| PI-E6 | Pricing, payment and incentive arrangements | Shapes service delivery through price policy, health-insurance payment, provider payment, emergency aid, reimbursement or performance-linked incentives. | Provider-payment, reimbursement, service-pricing, emergency-aid, or performance-linked financial arrangements with a clearly traceable application to stroke care or its service pathway. | Direct government investment or equipment procurement. General payment reforms without a clearly traceable relationship to stroke care or its service pathway were not coded. |

**Section: Policy instruments—demand-side**

| **Code** | **Operational label** | **Operational definition** | **Inclusion indicators** | **Key exclusion or boundary** |
| --- | --- | --- | --- | --- |
| PI-D1 | Screening, risk identification and early intervention | Directly expands or implements screening, risk assessment, early identification, early diagnosis or early intervention. | High-risk screening, risk assessment, early treatment and case finding. | General prevention statements without an operational screening/intervention action. May co-code PI-E1 when a quantified target is present. |
| PI-D2 | Public education, participation and self-management support | Activates or supports the public, patients or high-risk groups through education, participation, risk communication, self-assessment or self-management. | Health education, public mobilization, self-assessment and adherence/self-management support. | Internal professional training or administrative communication. Patients/residents are actors only when the text assigns them an active action; otherwise they are policy objects. |
| PI-D3 | Service access, referral and continuity | Extends access or links services across settings, levels or stages, including referral, follow-up, community/home services and continuity. | Two-way referral, post-discharge follow-up, community/home extension and service linkage. | Pure network construction without an explicit service-access or continuity component. Co-code PI-E2 when formal referral criteria are set; co-code PI-S1 when a service network is built. |

**Section: Governance tasks**

| **Code** | **Operational label** | **Operational definition** | **Inclusion indicators** | **Key exclusion or boundary** |
| --- | --- | --- | --- | --- |
| TK-01 | Risk-factor control and high-risk screening | Controls modifiable risks and identifies or intervenes in high-risk populations. | Risk assessment, hypertension/diabetes/AF control, screening and early intervention. | General health promotion with no risk-control or screening component. May co-code TK-07 when population education/health promotion is explicit. |
| TK-02 | Standardized diagnosis, treatment and acute care | Standardizes diagnosis/treatment or improves time-sensitive acute stroke care. | Clinical pathways, green channels, emergency treatment, reperfusion and standardized diagnosis/treatment. | General quality governance without clinical-care content. Acute emergency-system organization may additionally receive TK-06. |
| TK-03 | Stroke-center development | Creates, manages, designates or strengthens stroke centers and their regional roles. | Stroke-center construction, management, certification, training and regional-platform functions. | General hospital or specialty construction without a stroke-center component. Quality-control work inside stroke centers may also receive TK-05. |
| TK-04 | System building and coordination | Builds cross-level, cross-sector or regional systems and coordination mechanisms. | Medical alliances, emergency networks, hierarchical systems and actor coordination. | Single-institution service provision without system linkage. May co-code TK-06 when the system explicitly spans rehabilitation/continuity. |
| TK-05 | Quality control and performance improvement | Improves quality, safety, performance or compliance through monitoring, indicators, evaluation or quality-control structures. | Quality-control centers, indicators, evaluation and performance improvement. | Routine data reporting alone. Clinical-standardization content may also receive TK-02. |
| TK-06 | Rehabilitation and continuity of care | Organizes rehabilitation, post-acute care, follow-up, community/home services or continuity across the care pathway. | Staged rehabilitation, referral to rehabilitation, follow-up and home/community rehabilitation. | Acute treatment only. Co-code TK-04 when continuity is organized through a system/network. |
| TK-07 | Population prevention and health promotion | Promotes population-level prevention, public awareness, healthy behavior and health literacy. | Public education, lifestyle promotion, mobilization and population-facing prevention. | Professional training or individual clinical treatment only. May co-code TK-01 when risk screening/control is also explicit. |

**Section: Responsible actors**

| **Code** | **Operational label** | **Operational definition** | **Inclusion indicators** | **Key exclusion or boundary** |
| --- | --- | --- | --- | --- |
| ACT-01 | Central administrative authorities | Central ministries, commissions, State Council bodies or national administrative authorities assigned an implementation, coordination or supervisory role. | National Health Commission, State Council departments and central administrative offices when a role is assigned. | Document issuer listed only in the header. Use only for an action-bearing actor in the unit or permitted context window. |
| ACT-02 | Local health authorities | Provincial, municipal, county or territorial health authorities assigned a role. | Provincial health authorities and health administrative departments at lower territorial levels. | A generic territorial phrase when no administrative role can be established from context. Territorial phrases directing local implementation are coded only when the surrounding section clearly assigns responsibility to territorial authorities. |
| ACT-03 | Hospitals, medical institutions and stroke centers | Hospitals, base hospitals, medical institutions or stroke centers assigned a clinical, managerial, training or coordinating role. | Tertiary hospitals, base hospitals, stroke centers, and other medical institutions. | Institution named only as a destination/beneficiary. A named hospital or center is an actor only when it receives an explicit action predicate. Institutions embedded in names of formal arrangements, or mentioned only as expected to play a role, remain objects unless independently assigned an action. |
| ACT-04 | Primary healthcare and public-health institutions | Community health centers, township hospitals, family-doctor teams, CDCs or public-health institutions assigned a role. | Primary healthcare institutions, community health service institutions, township health centers, and disease-control institutions. | Population groups or general community settings. A primary-care/public-health institution directly followed by an action predicate is a strict actor; a referral destination without an assigned action remains an object. |
| ACT-05 | Emergency medical providers | Emergency centers, ambulance systems, dispatch centers or emergency departments assigned an action. | Emergency centers or stations, prehospital emergency medical institutions, and emergency departments. | Emergency care as a policy domain without a named responsible provider. Do not infer ACT-05 from a heading referring to prehospital emergency care alone. |
| ACT-06 | Rehabilitation providers | Rehabilitation hospitals, rehabilitation departments, rehabilitation centers or rehabilitation professionals assigned an action. | Rehabilitation hospitals, rehabilitation departments, and rehabilitation medical institutions. | Rehabilitation as a destination only. Destination-only mentions are policy objects; action-bearing providers are actors. |
| ACT-07 | Quality-control, technical and expert bodies | Quality-control centers, technical lead units, expert committees or professional technical organizations assigned a role. | Quality-control centers, expert committees, and technical guidance organizations. | Hospitals merely performing internal quality work. Use ACT-03 for hospitals unless they are explicitly designated as a regional technical/quality body. |
| ACT-08 | Other governmental departments and supporting organizations | Non-health government departments, insurers, education bodies, civil-affairs bodies, disability organizations and similar supporting actors. | Health-insurance, development and reform, finance, education, public security, transport, disability, and other departments when assigned a role. | Joint issuer lists without role allocation. When several supporting departments were explicitly assigned distinct roles, all applicable actor assignments were retained. |
| ACT-09 | Patients, residents and high-risk persons as active actors | Patients, residents or high-risk persons explicitly required or encouraged to perform an action such as self-assessment, self-management or adherence. | Resident self-assessment, patient adherence, self-management and active participation. | Being screened, treated, or educated without an assigned action. Assign ACT-09 only when the population group is explicitly required or encouraged to perform an action; passive recipients remain policy objects. |
| ACT-10 | Territorial/local governments | Local governments or territorial jurisdictions assigned an action when no functional department is identified. | Explicit action-bearing territorial phrases directing local or regional implementation, or an explicit construction instructing localities to act. | Geographic locations used only as settings; named local health authorities, which remain ACT-02. Do not infer ACT-10 from a location used only as a setting. A territorial expression is coded as an actor only when it is syntactically linked to an implementation action. |

**Section: Policy objects**

| **Code** | **Operational label** | **Operational definition** | **Inclusion indicators** | **Key exclusion or boundary** |
| --- | --- | --- | --- | --- |
| OBJ-01 | General population/residents | Residents, the public or general population targeted by policy actions. | Public education, general prevention and public emergency training targets. | Named high-risk or patient groups. Can coexist with ACT-09 only when the same population is also assigned an active action. |
| OBJ-02 | High-risk populations | People at elevated stroke/cerebrovascular risk or with defined risk factors. | High-risk screening and intervention populations. | Confirmed stroke/TIA patients when the policy action concerns treatment or secondary prevention. Use both OBJ-02 and OBJ-03 only when both groups are explicitly addressed. |
| OBJ-03 | Stroke/TIA/cerebrovascular patients | Patients with stroke, TIA or other cerebrovascular disease. | Acute treatment, rehabilitation, secondary prevention and follow-up populations. | General high-risk populations without diagnosed disease. Patient is an object unless an active responsibility is assigned. |
| OBJ-04 | Hospitals and stroke centers | Hospitals or stroke centers targeted for construction, regulation, assessment or capacity improvement. | Stroke-center construction, hospital quality assessment and designation. | Hospitals assigned an active role in the same unit. An institution may be both actor and object when the text assigns action and regulates/builds it. |
| OBJ-05 | Primary-care/public-health institutions | Primary-care or public-health institutions targeted for capacity building, referral or service expansion. | Community health centers, township hospitals, CDCs as targets. | Institutions explicitly assigned an action only. May coexist with ACT-04. |
| OBJ-06 | Emergency-care system/providers | Emergency networks, centers, ambulances or emergency services targeted for construction, regulation or improvement. | Emergency-network construction and quality improvement. | Emergency provider assigned an active action only. May coexist with ACT-05. |
| OBJ-07 | Rehabilitation system/providers | Rehabilitation services, networks or providers targeted for development, referral or payment support. | Rehabilitation hospitals, networks, community/home rehab and rehab payment. | Rehabilitation provider assigned an action only. May coexist with ACT-06. |
| OBJ-08 | Health workforce | Clinicians, emergency staff, rehabilitation professionals, family doctors or other personnel targeted for training or incentives. | Training, staffing, professional development and performance incentives. | Organizations rather than personnel. Use the most specific workforce category supported by the text. |
| OBJ-09 | Health-system/data/quality infrastructure | Systems, data platforms, quality structures or governance infrastructure targeted by policy action. | Information systems, quality-control systems and governance mechanisms. | Specific hospitals, patient groups or service providers. Use the most concrete object available; avoid OBJ-09 when a narrower code applies. |

**Section: Policy-integration domains**

| **Code** | **Operational label** | **Operational definition** | **Inclusion indicators** | **Key exclusion or boundary** |
| --- | --- | --- | --- | --- |
| INT-01 | Chronic disease governance | Links stroke/cerebrovascular policy to broader chronic-disease prevention and long-term risk management. | Chronic disease plans, hypertension/diabetes management and long-term risk control. | Acute care only. Record evidence units; assign the domain to the document only when at least one eligible unit is present. |
| INT-02 | Emergency care | Links stroke governance to prehospital emergency, dispatch, emergency departments or acute rescue systems. | Emergency networks, ambulances, green channels and prehospital-hospital linkage. | Non-urgent routine referral. A document may have both INT-02 and INT-04. |
| INT-03 | Rehabilitation and continuity | Links policy to rehabilitation, post-acute care, follow-up, home/community care or continuity. | Rehabilitation networks, staged referral and follow-up. | Acute treatment without continuity. Document-level assignment requires traceable unit evidence. |
| INT-04 | Hierarchical care and referral | Links stroke-related care to hierarchical diagnosis/treatment, two-way referral or level-based service roles. | Referral standards, medical alliances and tiered service provision. | Single-level treatment with no linkage. May co-occur with INT-03. |
| INT-05 | Specialty and stroke-center capacity | Links policy to stroke centers, neurological specialties, regional technical capacity or specialty development. | Stroke-center construction and specialty-capacity plans. | General hospital capacity with no specialty/stroke connection. Use only with explicit specialty or stroke-center evidence. |
| INT-06 | Public health and population prevention | Links policy to population prevention, community screening, health promotion or public-health systems. | Community screening, risk-factor surveillance and population education. | Hospital-only treatment. May co-occur with INT-01. |
| INT-07 | Medical quality and safety | Links policy to quality governance, safety, indicators, quality-control centers or performance improvement. | Quality control, safety, assessment and quality targets. | Routine service delivery without a quality mechanism. Document-level assignment requires evidence, not title alone. |
| INT-08 | Health financing and payment | Links policy to direct public/fiscal investment, service pricing, insurance/provider payment, emergency aid or other explicit financial mechanisms. | Government investment, earmarked/public funding, reimbursement, prices, provider payment, emergency aid and performance-linked financial arrangements with a pathway link. | Generic financing or payment reforms without traceable relevance to stroke care or its service pathway were not assigned to this domain. Direct investment is INT-08 but remains PI-S5 at the instrument level; pricing and payment arrangements are generally PI-E6. |

**** Document functions were assigned at the policy level; all remaining dimensions were coded at the atomic meaning-unit level. Responsible actors were recorded separately as strict and contextual assignments, and policy objects were coded separately from responsible actors. Codes were non-exclusive when more than one category had independent textual support, and no code was assigned when the relevant dimension was absent. Up to two document functions were retained when a policy had clearly separable principal roles.

**Appendix Table A8. Policy-level sensitivity analysis of stage differences in major policy-instrument composition.**

| **Analysis** | **Data representation** | **Statistic** | **Value** | **R-squared** | **P value** | **Holm-adjusted P** |
| --- | --- | --- | --- | --- | --- | --- |
| Global policy-level composition test | Centered log-ratio composition; 0.5 pseudocount | Pseudo-F | 1.899 | 0.112 | 0.147 |  |
| Global sensitivity analysis | Untransformed within-policy proportions | Pseudo-F | 2.174 | 0.127 | 0.089 |  |
| Multivariate dispersion check | Distance to stage centroid in CLR space | F | 1.744 |  | 0.183 |  |
| Supply-side policy-level proportion | Untransformed within-policy proportion | Kruskal-Wallis H | 3.238 |  | 0.198 | 0.198 |
| Environment-side policy-level proportion | Untransformed within-policy proportion | Kruskal-Wallis H | 6.639 |  | 0.036 | 0.109 |
| Demand-side policy-level proportion | Untransformed within-policy proportion | Kruskal-Wallis H | 5.086 |  | 0.079 | 0.157 |

**** The 33 policies were treated as independent analytical units. The primary test used centered log-ratio transformed within-policy compositions after adding a 0.5 pseudocount and 99,999 stage-label permutations (seed 20260701). R-squared is the proportion of compositional variation explained by stage. The raw-proportion analysis and dispersion test assessed robustness. Category-specific Kruskal-Wallis tests were adjusted across the three instrument categories using the Holm method. No post hoc stage-pair comparisons were performed because the global test was not statistically significant. Assignment-level chi-square testing was not used because multi-label assignments were nested within meaning units and policies.
